# A kinetic mechanism for enhanced selectivity of membrane transport

**DOI:** 10.1371/journal.pcbi.1007789

**Published:** 2020-07-02

**Authors:** Paola Bisignano, Michael A. Lee, August George, Daniel M. Zuckerman, Michael Grabe, John M. Rosenberg

**Affiliations:** 1 Cardiovascular Research Institute, Department of Pharmaceutical Chemistry, University of California San Francisco, San Francisco, California, United States of America; 2 Department of Biomedical Engineering, Oregon Health and Science University, Portland, Oregon, United States of America; 3 Department of Biological Sciences, University of Pittsburgh, Pittsburgh, Pennsylvania, United States of America; Icahn School of Medicine at Mount Sinai, UNITED STATES

## Abstract

Membrane transport is generally thought to occur via an alternating access mechanism in which the transporter adopts at least two states, accessible from two different sides of the membrane to exchange substrates from the extracellular environment and the cytoplasm or from the cytoplasm and the intracellular matrix of the organelles (only in eukaryotes). In recent years, a number of high resolution structures have supported this general framework for a wide class of transport molecules, although additional states along the transport pathway are emerging as critically important. Given that substrate binding is often weak in order to enhance overall transport rates, there exists the distinct possibility that transporters may transport the incorrect substrate. This is certainly the case for many pharmaceutical compounds that are absorbed in the gut or cross the blood brain barrier through endogenous transporters. Docking studies on the bacterial sugar transporter vSGLT reveal that many highly toxic compounds are compatible with binding to the orthosteric site, further motivating the selective pressure for additional modes of selectivity. Motivated by recent work in which we observed failed substrate delivery in a molecular dynamics simulation where the energized ion still goes down its concentration gradient, we hypothesize that some transporters evolved to harness this ‘slip’ mechanism to increase substrate selectivity and reduce the uptake of toxic molecules. Here, we test this idea by constructing and exploring a kinetic transport model that includes a slip pathway. While slip reduces the overall productive flux, when coupled with a second toxic molecule that is more prone to slippage, the overall substrate selectivity dramatically increases, suppressing the accumulation of the incorrect compound. We show that the mathematical framework for increased substrate selectivity in our model is analogous to the classic proofreading mechanism originally proposed for tRNA synthase; however, because the transport cycle is reversible we identified conditions in which the selectivity is essentially infinite and incorrect substrates are exported from the cell in a ‘detoxification’ mode. The cellular consequences of proofreading and membrane slippage are discussed as well as the impact on future drug development.

## Author summary

There is an important group of proteins, secondary active transporters, that are integral parts of membranes and that act as molecular pumps to move specific molecules, often referred to as cargo, across those membranes. This is an active process that typically exploits the energy inherent in the ionic gradients cells maintain across their membranes. Many transporters bind their cargos weakly because this is a physical requirement for rapid transport and this creates a problem that they would then fail to discriminate between the correct cargo and chemically similar molecules that would have a harmful effect if transported. Here, we explore the hypothesis that this discrimination can be enhanced by biochemical processes known in the literature as “proofreading” and/or “editing”. This exploration includes the expansion of an established kinetic model of a known transporter from *V. parahaemolyticus* (vSGLT) that transports sugars; mathematical analysis of the expanded model; numerical solution of the differential equations at the core of the expanded model; and a combination of data-base searches and molecular docking studies that establish the existence of toxic molecules sufficiently similar to the target cargo that they could be transported by vSGLT. We conclude that proofreading/editing is a plausible addition to the repertoire of secondary active transporters that would resolve the discrimination conundrum described above. We also note that the specifics of the model reported here reveal an interesting aspect of proofreading/editing behavior (unbounded selectivity) that, to our knowledge, has not been reported previously.

## Introduction

Secondary active transporters exploit the electrochemical gradient of ions across the membrane to couple ionic movement with that of specific solutes (cargo). In the late 1960’s, Jardetzky proposed the well-known alternating access mechanism [1] in which the membrane transporter switches between different conformational states that alternatively expose the solute binding site to either side of the membrane. There is now a wealth of structural data supporting the alternating access hypothesis illustrating at the atomic level how different transporter architectures carry out this function [2–7]. This mechanism of ion and cargo movement is generally thought to be tightly coupled because weak coupling (usually referred to as slip or leak) would run down ion gradients potentially having a profound impact on cellular viability. Moreover, many secondary active transporters have relatively weak affinities for their cargo, typically in the high micromolar, low millimolar range [8–10]. This realization raises a question regarding the ability of such transporters to discriminate between the correct cargo and biochemically similar compounds that could be harmful or even toxic. For example, the sodium/iodide (NIS) transporter is known to transport perchlorate, among other compounds highly problematic in the thyroid [11], and GLUT2 glucose transporters transport streptozotocin, a glucose analog that is particularly toxic to insulin secreting beta cells [12]. Based on these examples and the known promiscuity of multidrug efflux pumps, we expect that transporters are often challenged and fail to discriminate their endogenous substrates from other molecules in the environment, including toxic compounds. For those transporters that bind their substrates weakly, such as the sodium/glucose symporters from *Vibrio parahaemolyticus* (vSGLT), which we focus on here, how can they avoid the uptake of toxic molecules? This concern is especially pressing for bacteria like *Vibrio*, a marine microbe, that co-exist with competitive organisms capable of excreting lethal compounds, potentially toxic analogs of glucose that could be imported via vSGLT. One potential answer to this question, is that transporters may employ a proofreading/editing mechanism to achieve enhanced selectivity from a weakly or moderately selective binding site [13–15]. All proofreading/editing mechanisms include branched pathways some of which lead to the ejection of the incorrect substrate from a protein or enzyme before it can be converted to the final product. This ejection forms the basis of the enhanced selectivity, but it comes at a price: The correct substrate is also ejected part of the time as was clearly demonstrated by Fersht’s analysis [13, 16]. It demonstrated a direct relationship between enhanced selectivity and the cost of futile enzymatic cycles [13–15].

**Fig 1:**
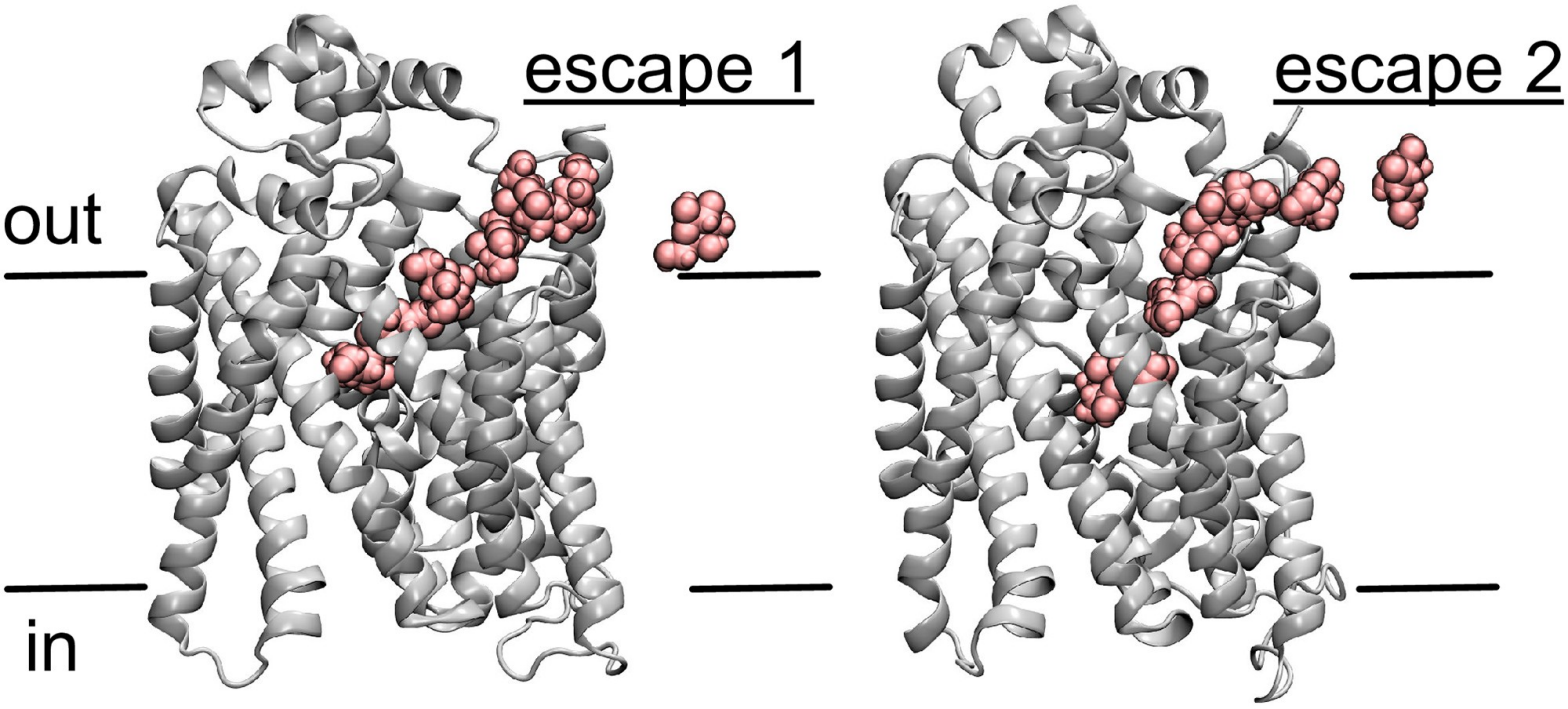
Substrate slippage event observed in molecular dynamics simulations. Molecular dynamics simulations performed on an inwardly oriented vSGLT structure revealed two unbinding events to the extracellular side, labeled escape 1 and 2. The protein is represented as cartoon, and the escaping galactose molecule (pink) is depicted in van der Waals over 7 snapshots along the escape pathway (data from Ref. [17]).

Just such an ejection of sugar was noted during molecular dynamics (MD) simulations [17] we previously carried out on the inward-facing conformation of vSGLT [18]. Specifically, the sugar, galactose in this case, was released outward through a partially-open outer gate in 2 of 21 simulations, or about 10% of the time (Fig 1). That is, a sugar which was initially placed in the crystallographic binding site was subsequently released to the extracellular side rather than the cytoplasmic release expected from an inward-facing state. This observation was the stimulus that lead us to apply the ideas of proofreading/editing to sugar symporters, as reported here. There are subtle differences between the descriptions of “kinetic proofreading” given by Hopfield and Ninio [14, 15] and the description of “editing” given by Fersht [13]. A clear, conceptual description of kinetic proofreading is given by Alon [19] while a similar description of editing is given by Fersht [16]. Kinetic proofreading ideas have inspired interesting applications, for example, Banerjee and co-workers recently investigated the interaction between speed and accuracy in a number of different contexts using ideas from kinetic proofreading [20]. Here, we present a kinetic model that incorporates ideas from both descriptions. Indeed, our model becomes identical to those of Fersht, Hopfield and Ninio by setting appropriate rate constants equal to zero.

In his original development of editing, as he terms it, Fersht made three basic assumptions [13, 16]:

1. The biochemical reaction pathway must be forked with one fork leading to the final product and the other fork leading back to the initial state without useful product. This unproductive fork results in an energetic cost to the system.
2. The correct substrate must take the wrong fork a fraction of the time. This represents the cost of proofreading.
3. The wrong substrate must take the unproductive fork a significantly larger fraction of the time than the correct substrate.

Fersht then went on to define cost, selectivity, and other quantities in terms of rate constants. His principal conclusion was expressed in the cost-selectivity equation, which showed that the enhanced selectivity intrinsically depended on the cost, i.e. there is no enhanced selectivity if the cost is zero. It is noteworthy that multiple steps in his model are irreversible and that his model is simpler than those necessary for secondary active transport.

**Fig 2:**
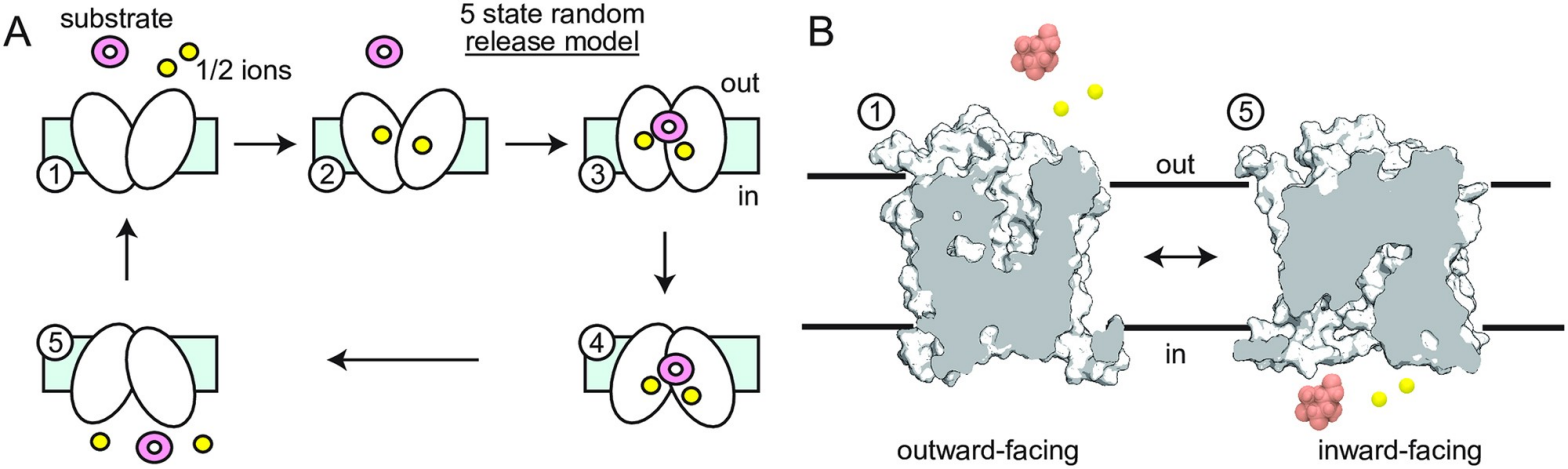
Kinetic models of sugar transport by SGLT symporters. **A)** Standard 5 state model of sodium-coupled transport based on Refs. [8, 17]. The protein cycles through outward empty (1), Na^+^-bound outward-open (2), substrate-bound occluded (3), substrate-bound inward-open (4), and inward-facing apo (5) states. Sugar (pink) and Na^+^ (yellow) bind in an ordered manner from the extracellular side, but are randomly released to the cytoplasm. Two Na^+^ are represented to indicate that some SGLTs bind 2 ions per cycle (hSGLT1), while others bind 1 (vSGLT and hSGLT2). **B)** Surface slice and cartoon view of vSGLT outward- and inward-facing states before substrate binding and after substrate release, respectively.

Branched pathways with imperfect coupling have been analyzed theoretically in the transporter field for years with early work by Hill [21] and more recent models inspired by direct biophysical measurement such as work on LeuT by LeVine and co-workers [22] or the EmrE multi-drug efflux pump by the Henzler-Wildman lab [23]. However, these models have not explored the transport of two different substrates simultaneously, which is an essential element of kinetic proofreading. That said, Henzler-Wildman’s latest work showed that EmrE can switch from a symporter to an antiporter in a substrate dependent manner [23]. We start from a detailed kinetic model of the Na^+^-dependent sugar transport cycle for human SGLT1 based on quantitative electrophysiological recordings from the Wright lab [8, 17], and the most recent five state kinetic model is shown schematically in Fig 2. In this model, ions first bind from the extracellular side (1 for vSGLT and 2 for hSGLT1), followed by substrate binding. Next, the protein undergoes an isomerization to an inward-facing state followed by random release of either substrate or Na^+^ before the system resets to the outward facing state (state 5 to 1, pictured in atomic detail in panel B). Each step is reversible as these transporters can run forwards or backwards depending on the ionic gradients. However, there is no fork in this cycle, and it only considers the endongenous substrate—both required elements for proofreading. Here, we construct an additional transition inspired by our simulations that provides this forked step as sugar is delivered to the cytoplasm or ejected to the extracellular space from an inward-facing conformation. The state space is then mirrored to include toxin binding states, and we derive equations for cost and selectivity to compare with classic formulations from the literature. Next, we explore the role of kinetic rates on the full transport cycle, flux values, and selectivity by solving for the steady state solutions of the resulting kinetic equations. Our analysis reveals a unique gain of selectivity that arises from the reversibility of these transporter systems coupled with ion and cargo gradients, which to the best of our knowledge, has not been previously identified.

## Results and discussion

### A kinetic slip model based on violation of alternating access

Based on our previously reported observation of sugar escape to the extracellular space during simulations of an inward-facing conformation [17] (also see Fig 1), vSGLT appears to violate the assumptions of strict alternating access for the sugar substrate a small fraction of the time, as hypothesized for the Mhp1 Na^+^-dependent transporter based on coarse-grained simulations [24]. These sugar escapes are consistent with previous simulations revealing transient water conducting states in vSGLT and other LeuT family members [25–27], while direct experimental evidence exists for partial outer gate uncoupling in LeuT based on single molecule FRET data [28]. Experimental evidence of uncoupling between the gates does exist for the human homologue hSGLT1, as the Wright lab noted a leak of about 300 water molecules per transport cycle [29]. More generally, Poolman and colleagues noted that the slippage mechanism in membrane symporters (such as the bacterial proton/sugar symporters LacY, LacS, and Hup1, the eukaryotic proton/metal ion transporter DCT1, and the human proton/folate symporter PCFT) could be essential to prevent cell lysis from high osmotic pressure resulting from the excessive accumulation of substrates [30].

**Fig 3:**
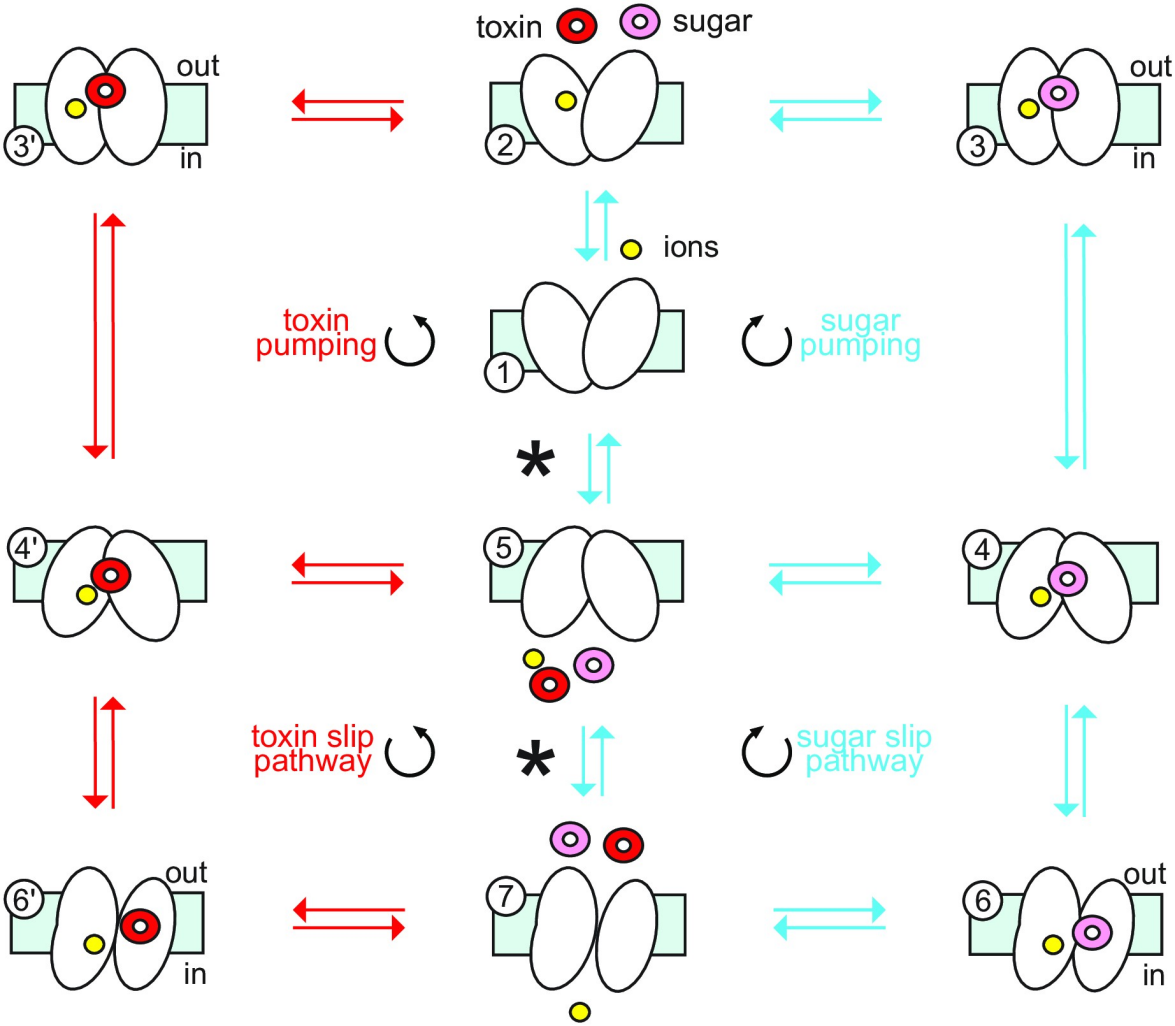
Kinetic model of substrate and toxin transport with slip. The slip mechanism, represented in the lower cycles, includes an inward-open conformation with a partially-open outer gate (states 6, 6’, 7) corresponding to states in Fig 1. Transitions from 6 or 6’ to 7 involve the Na^+^ entering the cell going down its energy gradient, while the toxin (red molecule) or sugar (pink) escape to the extracellular space. The right set of states (blue transitions) all involve sugar movement, the left states (red) involve toxin movement, while the upper states are based on the canonical pumping cycles in Fig 2A. All toxin transport states are identically mirrored based on corresponding sugar states, but rate constants have been modified as discussed in the text. Additionally, the full outer cycles on the right and left are Na^+^ leak cycles that move the ion down its gradient. Note that the 5 to 7 transition indicated by an asterisk is a conformational change that does not involve substrate movement. The cycles and pumping definitions for sugar are explained in S1 Fig.

With this in mind, we modified the canonical 5 state kinetic model in Fig 2 to include a slip state. This expanded scheme for the endogenous sugar substrate is shown on the right hand side of Fig 3 in blue, and the model acquires two more states, 6 and 7. These states represent the transporter in the inward-facing state with a partial open-outer gate observed in our simulations shown in Fig 1, and moving from state 6 to 7 involves the Na^+^ moving down its concentration gradient to the cytoplasm while the sugar fails to transport and moves to the extracellular space, consistent with our simulations [17]. State 4 of the scheme now becomes the decision fork essential in proofreading models by separating the sugar pumping cycle (4 → 5) from the sugar slip pathway (4 → 6).

Next, we included the transport of toxic sugar analogs in the model by mirroring the sugar cycles on the right (blue) in a one-to-one correspondence (primed states on the left connected with red arrows). As for the sugar, Na^+^-dependent toxin pumping into the cell occurs along the toxin pumping cycle (1 →2→ 3’ → 4’ → 5), while toxin slip occurs along the slip pathway (4’ → 6’ →7→ 5). As discussed in the Model and Methods and Supporting Information, we formulated the Master Equations corresponding to the schematic in Fig 3 and solved for the steady state solution under different conditions.

### An editing/proofreading model for secondary active transporters

Fersht derived his expressions for enhanced selectivity, including the cost-selectivity equation, in terms of rate constants that included irreversible steps [13, 16]. Here, we recast these ideas for reversible transport including slip by focusing on flows through the system rather than specific rate constants. There are three key fluxes in our model: the total binding flux of substrates from the extracellular space ($J^{bind}$), the total productive pumping flux ($J^{pump}$), and the rate of substrate slip ($J^{slip}$). Additionally, separate flows exist for sugar (subscript S) and toxin (subscript W). The fluxes can be determined from the scheme in Fig 3 by solving the corresponding kinetic equations. The pumping flux of sugar is given by the 4 → 5 transition in which sugar is released to the cytoplasm from the inward-facings state, and the corresponding pumping flux of toxin is given by the 4′ → 5 transition. Similarly, flow for the slip dissociation to the extracellular side occurs only along one transition—6 → 7 for sugar and 6′ → 7 for toxin. In both of these cases, the transporter has adopted an inward-facing conformation with a partially-open outer gate, and the substrate escapes to the extracellular space but the Na^+^ goes down its concentration gradient wasting energy. Finally, the total binding flux from the extracellular space is again given by only a single transition, which involves substrates binding to the orthosteric site from the outside (2 → 3 for sugar and 2 → 3′ for toxin). At steady state, conservation of mass relates these three quantities because the productive flux of pumped substrate into the cell must be equal to the binding flux to the transporter minus the slip flux:

$$\begin{array}{c} J_{X}^{pump}=J_{X}^{bind}-J_{X}^{slip}, \end{array}$$ (1)

where *X* can be S for sugar or W for toxin. Importantly, because the model is reversible, these fluxes can be negative.

With these flows defined, the selectivity of transport (*σ*) at steady state is related to the ratio of the productive sugar pumping rate to productive toxin pumping rate:

$$\begin{array}{ccc} \frac{J_{S}^{pump}}{J_{W}^{pump}} & = & \sigma \frac{\Delta [S]}{\Delta [W]}, \end{array}$$ (2)

where Δ[S] and Δ[W] are the concentration differences or driving forces for sugar and toxin flow across the membrane, respectively, and we use the symbol *σ* rather than S, as used by Fersht, due to the extensive use of the letter S here. With this definition, if the driving forces for each substrate are equal, the selectivity is simply the ratio of the number of sugar events observed per unit time divided by the number of toxin events, and *σ* will be 1 for a non-selective transporter and *σ* ≫ 1 for a highly selective transporter. In general, the flux of a given molecule depends on its driving force; and therefore, the selectivity equation also incorporates the ratio of the concentration differences to account for this bias.

Next, if there is a price for enhanced selectivity then what is the measure of that price? One answer given by Fersht is that it is essentially the fractional rate of false positive errors [16], i.e. the rate of rejection of valid substrate, which is sugar in this case, divided by its binding flux. We will use Fersht’s terminology as our working definition of ‘cost’ throughout, but we acknowledge that alternative measures are possible. While it appears that there is no cost for sugar to slip back to the extracellular space, in fact every such cycle involves a Na^+^ ion moving down its energy gradient from the outside to the inside of the cell. Specifically, we define that fractional rate as the cost (C) which can be stated in terms of flows as follows:

$$\begin{array}{ccc} C & \equiv & \frac{J_{S}^{slip}}{J_{S}^{bind}}=\frac{J_{S}^{slip}}{J_{S}^{pump}+J_{S}^{slip}}, \end{array}$$ (3)

where again the formal definition is general and applies away from steady state, while the ratio on the right hand side only applies at steady state. If the slip goes to zero, there is no cost associated with the transport cycle (C=0) because every sugar that binds is successfully pumped. Conversely, if the pumping flux goes to zero while the slip remains finite, the cost is maximized at unity (C=1) because every slip event transports a Na^+^ down its concentration gradient without delivering a sugar to the cell.

With these concepts in hand, we derive in the Supporting Information (S1 and S2 Text) the cost-selectivity relationship for this system:

$$\begin{array}{ccc} \sigma & = & f[1+\left(f^{\prime }f^{\prime \prime }-1\right)C]\\ \sigma & = & \underset{f}{\underset{︸}{\frac{J_{S}^{bind}}{J_{W}^{bind}}\frac{\Delta [W]}{\Delta [S]}}}[1+(\underset{f^{\prime }}{\underset{︸}{\frac{J_{S}^{pump}}{J_{W}^{pump}}}}\cdot \underset{f^{\prime \prime }}{\underset{︸}{\frac{J_{W}^{slip}}{J_{S}^{slip}}}}-1)C], \end{array}$$ (4)

where the first line takes on the same form as Fersht’s cost-selectivity equation [16], and the terms *f*, *f*′, and *f*″ are defined in the second line as derived in the Supporting Information for this fully reversible system. The importance of the cost-selectivity equation is that it clearly shows when the cost is zero the maximum achievable selectivity is the basal level *f*, which is related to the binding flux of the correct substrate over the incorrect one. However, for C>0 it is possible to increase the selectivity, proportional to C, above this basal level for appropriate values of *f*′ and *f*″. For instance, as long as the sugar pumping rate is greater than the toxin pump rate (*f*′ > 1) and the toxin slips more readily than the sugar (*f*″ > 1) then the selectivity is enhanced by a multiplicative factor above the basal value. It is this exciting realization that inspired us to more fully explore this model numerically, since the additional complexity of the state space makes analytic analysis cumbersome and difficult to interpret. Next, we show that our model of transport with slip has the expected cost-selectivity profile; however, the reversibility inherent in transport systems gives rise to novel features that, to our knowledge, have gone unappreciated in the proofreading literature.

### Transport slip pathways can dramatically increase substrate selectivity

We carried out a series of calculations to determine if the addition of slip in the kinetic pathway increased the transporter’s ability to select against poorly discriminated substrates. First, we investigated the impact of slip and membrane potential on selectivity under conditions where the only discrimination between sugar and toxin was achieved by slowing the toxin binding and unbinding transition from occluded to inward facing (*k*_3′4′_) and back again (*k*_4′3′_) compared to sugar (Fig 3), and this change corresponded to a 1*k*_*B*_*T* increase in the barrier height compared to sugar ($\Delta \Delta G_{3^{\prime }4^{\prime }}^{‡}=1\;k_{B}T$).

The binding affinities of sugar and toxin were identical (see S1 Table). Unless otherwise noted, the Na^+^ concentrations were held constant at 125 mM extracellularly and 40 mM on the cytoplasmic side as were the sugar and toxin concentrations, which were fixed at 1 mM on the extracellular side and 4 mM on the cytoplasmic side. In all calculations, we determine all relevant quantities for both the full model with slip and the model with the slip pathways disabled (rate constants set to zero) as a control to assess how slippage increases selectivity, and detailed balance is explicitly enforced through the rate constants as shown in Eq 9.

**Fig 4:**
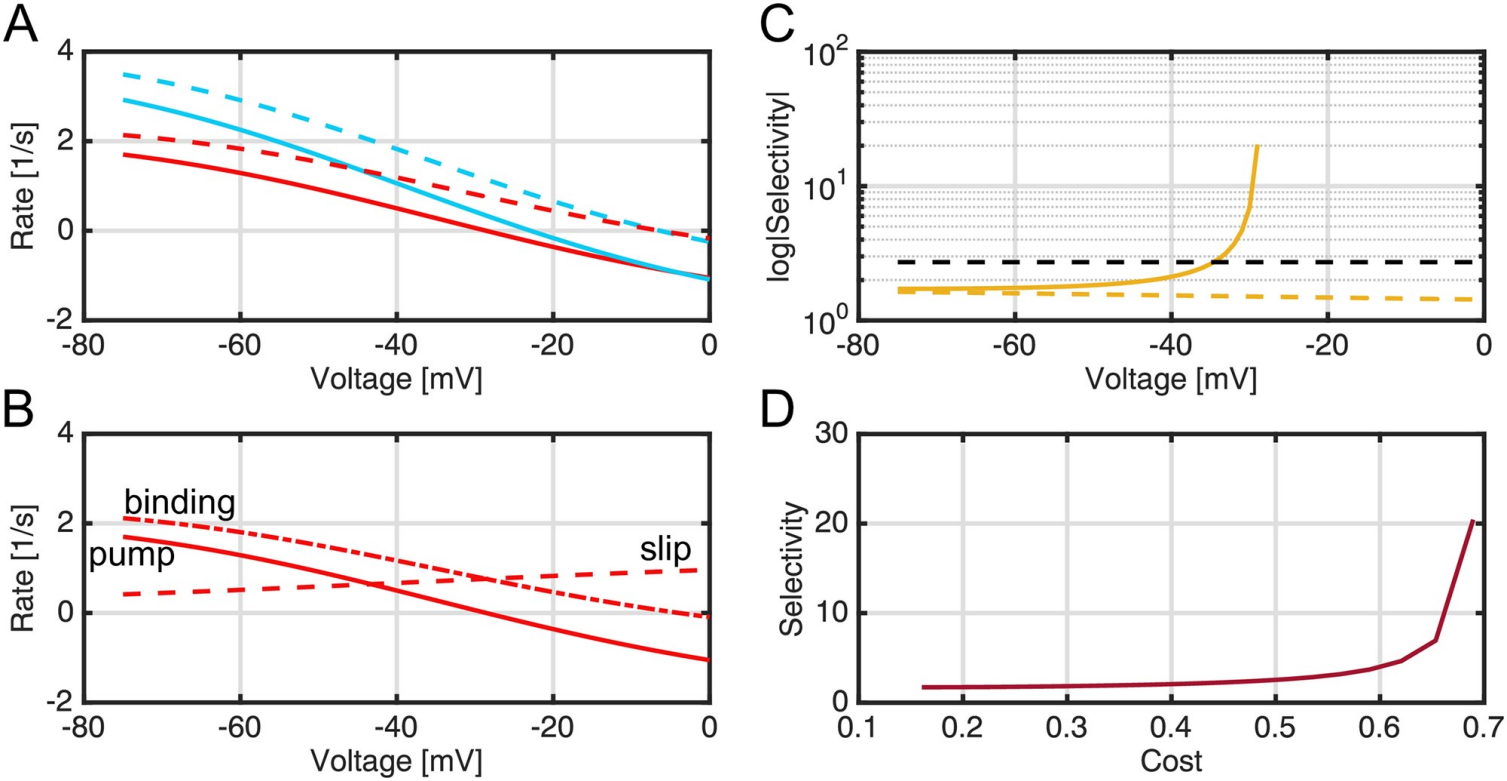
Flows, selectivity, and cost of a transport model with slip and weak discrimination during transport. For all calculations, the sugar binding transition to the inward state is 1 *k*_*B*_*T* more favorable than toxin ($\Delta \Delta G_{3^{\prime }4^{\prime }}^{‡}=1$), but all other parameters for sugar and toxin are identical as in S1–S3 Tables. **A)** The flows of sugar (blue) and toxin (red) into the cytoplasm in the presence (solid curves) and absence (dashed curves) of slippage plotted as a function of membrane potential. **B)** Decomposition of the toxin pumping cycle. Toxin pumping into the cytoplasm, $J_{W}^{pump}$, (solid curve) is equal to the rate of binding to the outward-facing conformation, $J_{W}^{bind}$, (dot-dash curve) minus the rate of toxin escape to the extracellular space, $J_{W}^{slip}$ (dashed curve) according to Eq 1. **C)** The selectivity (*σ*) as a function of membrane potential. Note that the ordinate (selectivity) is logarithmic. The solid gold curve is the selectivity calculated in the presence of slip (Eq 2), and the dashed gold line is the selectivity observed when slip is disabled. The dashed black line is the theoretical selectivity estimate based on a discrimination of 1 *k*_*B*_*T* at the inward-transition step. **D)** The selectivity (*σ*) is plotted versus cost (C, see Eqs 2–4).

First, we swept through physiological membrane potential values to alter the Na^+^ driving force, and we see that the sugar flow (blue dashed curve) was greater than the toxin flow (red dashed curve) in the absence of slippage due to the initial selectivity at the inward transition step (Fig 4A). Both flows monotonically decrease as voltage increases due to diminished Na^+^ driving force until they cross zero at thermodynamic stall (about -5 mV), which is the same for both molecules under these conditions. There is a slight efflux at 0 mV, consistent with the well-known reversibility of secondary active transporters, which is preserved in this kinetic model. The behavior in the presence of slip was notably different in two respects. First, the toxin flux (red solid curve) went to zero at a more negative voltage than the sugar flux (blue solid curve)—-30 mV versus -22 mV, respectively. Second, both sugar and toxin show significant efflux (negative rates) to the right of their respective crossing points. The voltage range between -30 and -22 mV defines a region, which we call the *detoxification regime*, in which toxin is flowing out of the cell while sugar is still being pumped in. The crossing points, one for toxin and one for sugar, represent dynamic steady states where sodium is flowing into the cell even though there is no net flow of the respective molecule. Nonetheless, toxin (and sugar) are still both flowing through the system at these points, but the the rate of extracellular toxin binding (2 → 3′ step, Fig 3) is balanced by the toxin release rate at the 6′ → 7 step when $J_{W}^{pump}=0$, as can be seen in Eq 1 resulting in no net flux into the cell. Fig 4B shows the toxin movement in the presence of the slip pathway in more detail. Even under strong inward-driving conditions at negative voltages, not all the toxin that binds (dash dot curve) is pumped (solid curve) into the cell, as some of the bound toxin is slipped (dashed curve) leading to unproductive cycles. Mass conservation in Eq 1 explains why the slip flux equals the binding flux when the pumping rate is zero at -30 mV. For voltages greater than -30 mV, the toxin is leaving the cell (the pumping rate is negative), and importantly, it is flowing down its concentration gradient as the cytoplasmic concentration is 4 mM and the extracellular concentration is 1 mM. The crucial parameter of interest is the selectivity (*σ*) of the sugar over the toxin for the slip cycle given by Eq 2 and plotted in Fig 4C on a log scale (solid gold curve). Under strong driving conditions at -75 mV, the model with and without slip (dashed gold curve) provide the same, meager selectivity of about 1.1 sugar to toxin. This value is even lower than the maximum expected theoretical selectivity (black dashed line) from the 1 *k*_*B*_*T* discrimination at the transition step, i.e. *σ* ≃ *exp*(1) = 2.72, which can only be realized if the inward transition step is rate limiting in the cycle. Moving right towards smaller driving forces, *σ* grows for the slip cycle, but it remains constant in the absence of slip when the substrate and Na^+^ movement are tightly coupled. The selectivity eventually surpasses the theoretical maximum around -35 mV and sharply rises to 20 at -30 mV as *σ* becomes unbounded due to the toxin pumping flux $J_{W}^{pump}$ going to zero. The increase in selectivity is so sharp that it is not well resolved in our voltage sweep. Moreover, as the system enters the detoxification regime, *σ* becomes negative since $J_{W}^{pump}<0$, and it is no longer defined on a log axis. It is both surprising and impressive that a small energy difference of just 1 *k*_*B*_*T* can lead to infinite selectivity. How is this possible? We believe that a useful analogy is that of a transistor amplifier, in which small differences in input voltage result in large differences in output voltage. This amplification arises from subtle differences in input voltage that divert currents flowing through the transistor such that the output voltage changes significantly. Here, a subtle change in the inward transition energy alters the flow of substrate through the system. Near stall, both the toxin and sugar pumping fluxes go to zero, but this small energy difference sensitizes the toxin flux to the addition of slip, and as the driving force diminishes the toxin pumping halts first giving rise to infinite selectivity and a region of detoxification. Traditional proofreading and editing models [13, 14] cannot achieve perfect selectivity, because it is not fully reversible.

Nonetheless, selectivity comes at a cost, which is the false positive rate we saw in our initial vSGLT simulations in which the correct substrate is rejected (Fig 1). This trend can be seen in Fig 4D, which is a plot of selectivity (*σ*) versus cost (C) summarized by the cost-selectivity equation (Eq 4). The cost increases monotonically with membrane potential, rising from about 0.17 at -75 mV to approximately 0.7 near -30 mV (right edge of Fig 4D). The selectivity (red curve in Fig 4D) is approximately 2 when C=0.17, and it increases slowly until C≈0.6 beyond which it rapidly increases. Note that a cost ratio of 0.7 means that only 30% of the extracellular sugar that binds at the 2 → 3 transition is actually released to the cytoplasm, while 70% is slipped back to the extracellular compartment via the 6 → 7 step, which may seem wasteful; however, the advantage is that the transporter is now highly selective against incorrect substrates that differ in only subtle ways from the sugar.

### Enhanced selectivity is broadened with multi-point discrimination

**Fig 5:**
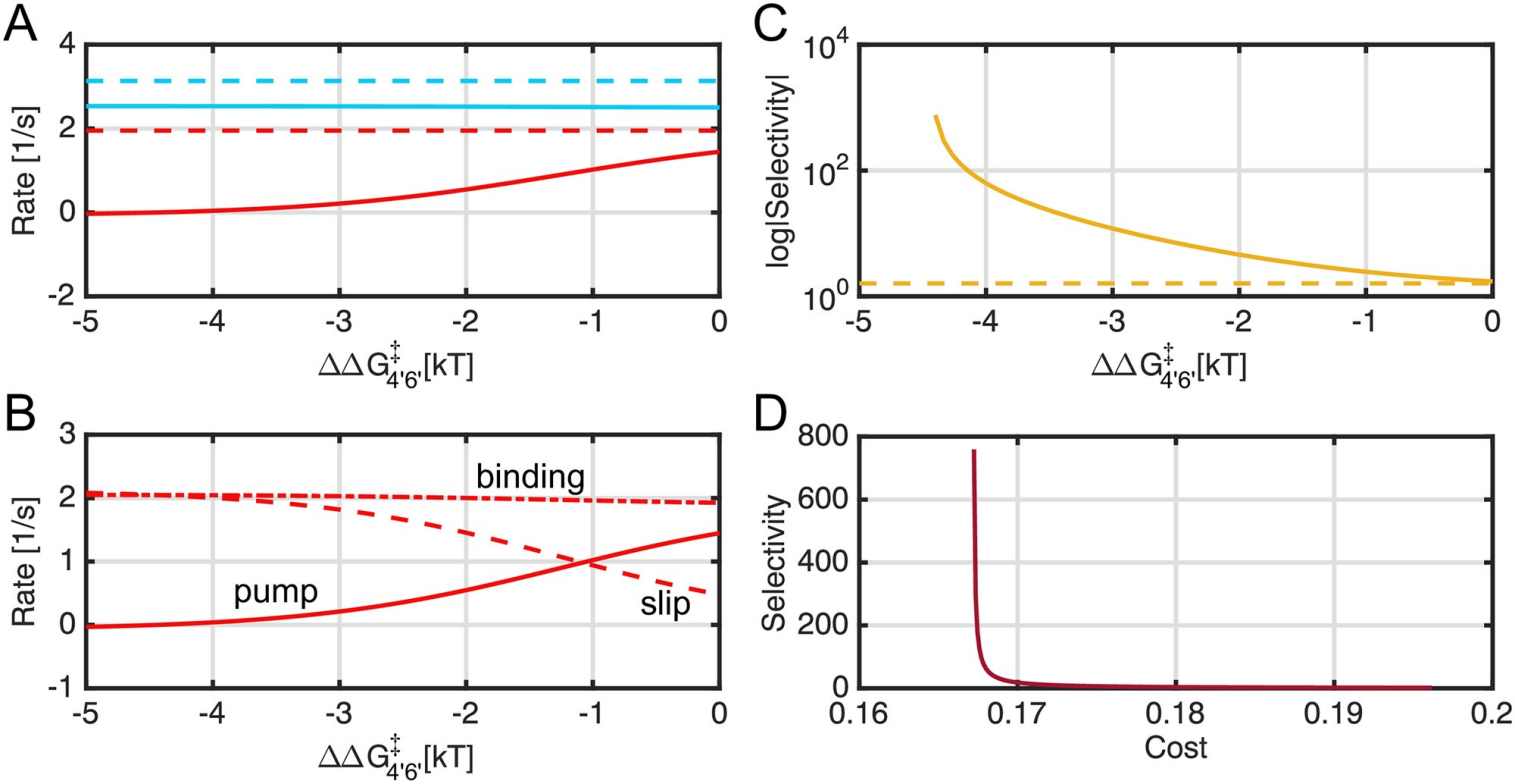
Flows, cost, and selectivity with discrimination added to the slip cycle. In addition to weak discrimination (1 *k*_*B*_*T*) at the inward-transition state, the rate of partial outer gate opening was modulated by changing $\Delta \Delta G_{4^{\prime }6^{\prime }}^{‡}$ from -5 to 0 *k*_*B*_*T*. **A)** Net sugar (blue) and toxin (red) pumping as a function of $\Delta \Delta G_{4^{\prime }6^{\prime }}^{‡}$ with (solid curves) and without (dashed curves) slip. Toxin import is reduced as outer gate opening becomes easier at negative values of $\Delta \Delta G_{4^{\prime }6^{\prime }}^{‡}$, but all other pumping fluxes are insensitive. Values at $\Delta \Delta G_{4^{\prime }6^{\prime }}^{‡}=0$ on right correspond to values at -75 mV in Fig 4A. **B)** Toxin pumping (solid), slipping (dashed), and binding (dash-dot) rates as a function of $\Delta \Delta G_{4^{\prime }6^{\prime }}^{‡}$. Note that the pumping flow of toxin is approximately zero when $\Delta \Delta G_{4^{\prime }6^{\prime }}^{‡}=$ -5 *k*_*B*_*T*. **C)** Selectivity (*σ*) as a function of $\Delta \Delta G_{4^{\prime }6^{\prime }}^{‡}$. The selectivity with slip is solid gold, and the selectivity with the slip cycle removed is dashed gold. **D)** Cost-selectivity curve as $\Delta \Delta G_{4^{\prime }6^{\prime }}^{‡}$ is varied in panels A-C. The cost (C) varies over a narrow range starting near 0.2 when $\Delta \Delta G_{4^{\prime }6^{\prime }}^{‡}=0$, and dropping below 0.17 as $\Delta \Delta G_{4^{\prime }6^{\prime }}^{‡}$ approaches -4.5 *k*_*B*_*T* at which point *σ* increases beyond 800. The membrane potential was held at -75 mV for all calculations.

Traditional proofreading/editing analyses emphasize the importance of discrimination at multiple steps [13, 14], in contrast to the single point of discrimination in the preceding discussion. While we saw that a single point of weak discrimination can attain unbounded selectivity, that required the system to be near stall where the pumping flux is small. We therefore introduced a second point of discrimination at the 4′ → 6′ step in the toxin slip cycle by varying $\Delta \Delta G_{4^{\prime }6^{\prime }}^{‡}$ while holding the voltage constant at -75 mV (see Fig 3), and while maintaining the 1 *k*_*B*_*T* of discrimination at the inward transition step. Mechanistically, state 4′ sits at the branch point between normal pumping and slipping, and $\Delta \Delta G_{4^{\prime }6^{\prime }}^{‡}$ modulates the rate of the outer gate’s (partial) opening and closing to allow slip from the inward-facing conformation. This was motivated by our obersvations of slip in the molecular dynamics simulations described above. As expected, the toxin pumping flow (solid red curve) decreased as $\Delta \Delta G_{4^{\prime }6^{\prime }}^{‡}$ became more negative (leftward in Fig 5A), while the sugar pumping flow (solid blue curve) was unaffected (Fig 5A). Both flows were constant when slippage was inhibited (dashed lines, blue—sugar, red—toxin), as expected since all slip cycle rates are set to zero for these calculations. When $\Delta \Delta G_{4^{\prime }6^{\prime }}^{‡}=0$, the toxin pumping rate (solid curve) is greater than the slip rate (dashed curve, Fig 5B), and the selectivity is low (Fig 5C). As the energy difference decreases (increasing the toxin slip rate), the toxin pumping decreases and the slip increases. Below -3 *k*_*B*_*T*, panel B reveals that the slip pathway dominates and the pumping rate approaches, and then crosses, zero around -4.5 *k*_*B*_*T*. As expected, over this range the selectivity (solid gold curve) grows above 100 until it reaches infinity as the toxin pumping halts (Fig 5C). For comparison, the tightly coupled no slip model (dashed gold curve) is essentially non-selective (Fig 5C).

Adding discrimination at a second step dramatically lowered the cost, which varied over a narrow range of 0.16 to 0.20 as $\Delta \Delta G_{4^{\prime }6^{\prime }}^{‡}$ was changed (Fig 5D). These values are more typical of those seen in Fersht’s analyses of DNA polymerase and threonine tRNA synthetase [16]; however, the cost-selectivity relationship is opposite to what we typically expect (increasing selectivity with increased cost) and what we observed in Fig 4. This occurs, primarily, because the energy landscape is being adjusted to increase ejection of the wrong substrate, which has a minor impact of decreasing the false positive rate (C) of the correct substrate. Here, the cost is essentially constant as the selectivity changes because the control parameter ($\Delta \Delta G_{4^{\prime }6^{\prime }}^{‡}$) effects the toxin ejection rate rather than the sugar, and these types of parameters are not typically varied in traditional proofreading analyses.

**Fig 6:**
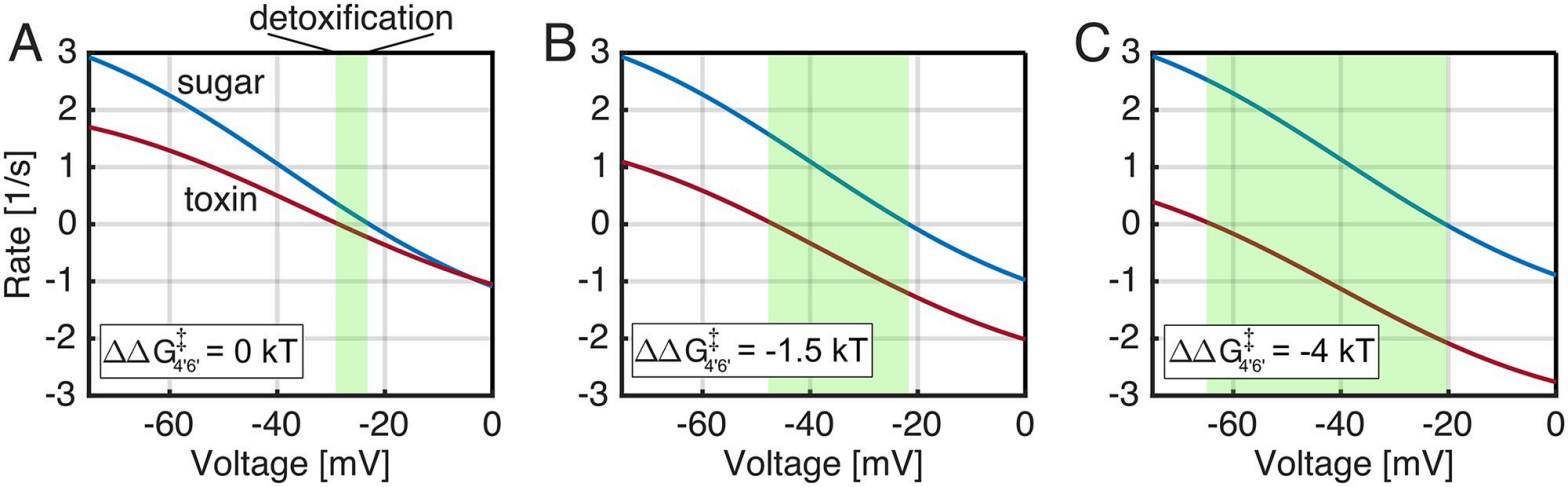
Two-point discrimination widens the detoxification regime. Toxin (red) and sugar (blue) pumping into the cytoplasm is plotted versus the membrane potential for $\Delta \Delta G_{4^{\prime }6^{\prime }}^{‡}$ = 0 **(A)**, -1.5 **(B)**, and -4 *k*_*B*_*T* **(C)**. The green regions indicate detoxification—negative toxin flow (export from cell) and positive sugar flow (into the cell). As with all other models, there is a 1 *k*_*B*_*T* discrimination at the transport step in addition to changes in panels B and C to the 4′ to 6′ step. The curve for 0 *k*_*B*_*T* is the same as the curve labeled “pumping” in Fig 4B.

Given the large impact on transport caused by altering the toxin slip pathway together with the initial discrimination of the transport step, we repeated the calculations in Fig 4 where we varied membrane potential while holding $\Delta \Delta G_{4^{\prime }6^{\prime }}^{‡}$ fixed at 0, -1.5, and -4 *k*_*B*_*T* (Fig 6). The toxin pumping rate ($J_{W}^{pump}$) decreases as $\Delta \Delta G_{4^{\prime }6^{\prime }}^{‡}$ is lowered resulting in pumping curves that cross the x-axis at more negative voltages $J_{W}^{pump}=0$ at -30, -50, and -65 mV when $\Delta \Delta G_{4^{\prime }6^{\prime }}^{‡}$ = 0, -1.5, and -4 *k*_*B*_*T*, respectively. As we saw before, when the toxin pumping rate goes to zero while sugar pumping is finite, the transporter achieves unbounded selectivity and enters the detoxification regime (green regions). These calculations show us that a two-point scheme can result in a detoxification regime over a broader range of voltages (-65 to -22 mV under these conditions) that are more physiological. Lastly, the cost-selectivity for $\Delta \Delta G_{4^{\prime }6^{\prime }}^{‡}=-4\;k_{B}T$ shows that a high selectivity value (5,000) can be obtained at more reasonable cost (0.17 or 83% of bound sugar is transported into the cytoplasm) than was noted in Fig 4 where $\Delta \Delta G_{4^{\prime }6^{\prime }}^{‡}=0$. This cost value is close to those in the classical editing literature [16].

### Not all leaks are created equal

Although the preceding discussion represents one way slip can be introduced into a strict alternating access mechanism, there other forms of non-standard alternating access [21–23, 30, 31]. Sodium slip is one of the more prominently discussed mechanisms for uncoupling [21], and it has been specifically discussed for LeuT-fold transporters like SGLT [22]. Therefore, we added a Na^+^-slip state to our model to allow an ion-only leak across the membrane by adding an inward-facing Na^+^-only bound conformation (state 8 in S2 Fig) connected to the outward-facing state 2 via rates *k*_28_ and *k*_82_ (see Supporting Information S1 Text for mathematical details). This model adds an independent Na^+^-slip in the absence of any substrate binding (1 → 2 → 8 → 5 → 1) enabling us to ask what happens when we completely decouple sodium transport from substrate binding.

**Fig 7:**
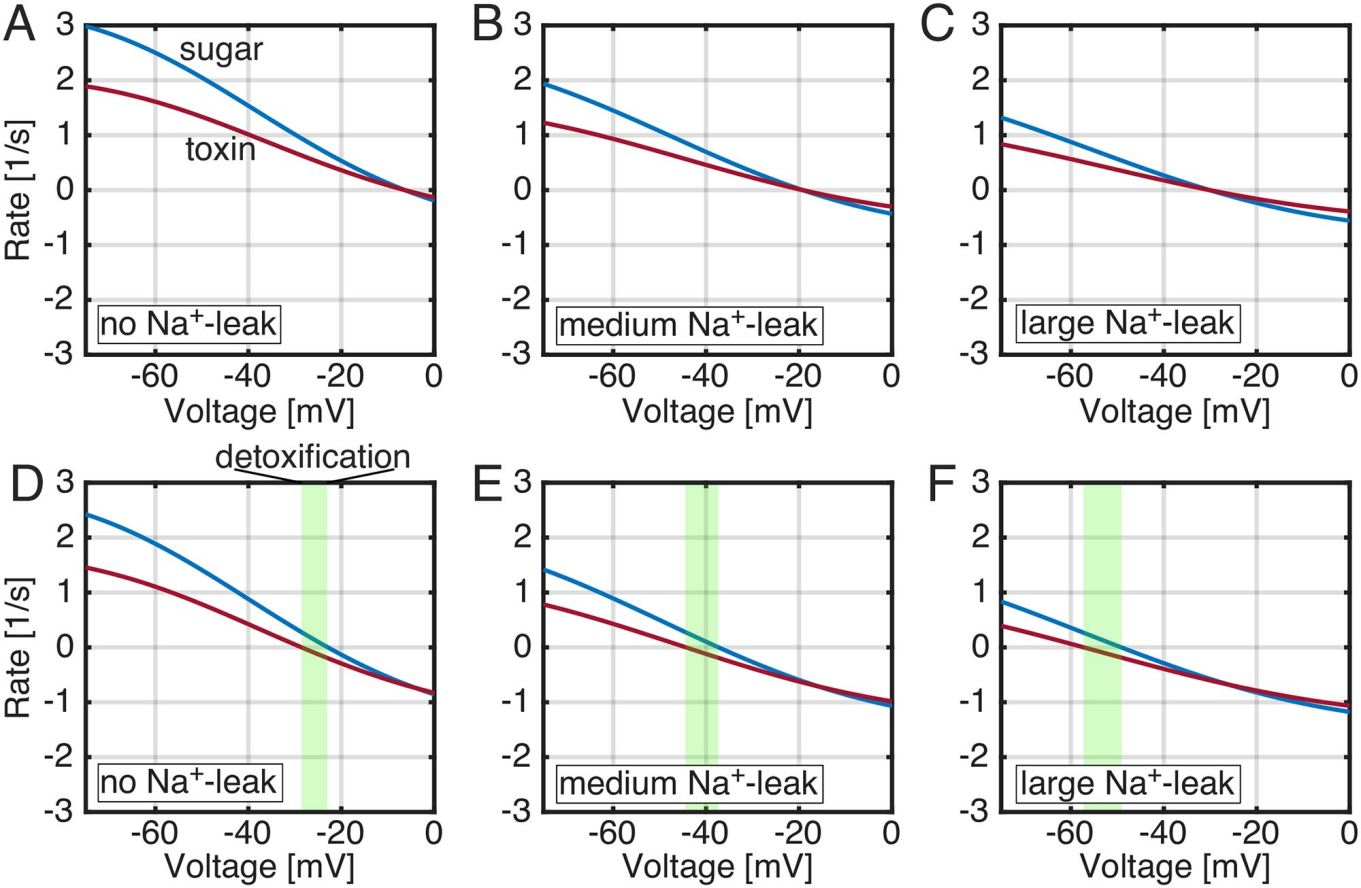
Flow of sugar and toxin in the presence of an independent sodium leak. The sugar (blue) and toxin (red) flows in the presence of varying levels of Na^+^-leak using the modified kinetic scheme in S2 Fig. **A-C)** The model was solved with no sugar slip and forward *k*_28_ Na^+^-leak values of 0 **(A)**, 100 s^−1^ **(B)**, or 200 s^−1^ **(C)**. The only discrimination between sugar and toxin is 1 *k*_*B*_*T* on the 3 → 4 step i.e. $\Delta \Delta G_{3^{\prime }4^{\prime }}^{‡}=1$ as in Fig 4. The curves in panel **A** are identical to the corresponding curves in Fig 4A. **D-F)** The full model was solved with sugar slip and increasing levels of Na^+^-leak: 0 **(D)**, 100 s^−1^ **(E)**, or 200 s^−1^ **(F)**. The curves in panel **D** are identical the solid curves in Fig 4A.

The consequences of the introduction of uncoupled, Na^+^-slip can be seen in Fig 7 where the principal takeaway is that Na^+^-slip alone fails to provide enhanced selectivity (panels A-C). Additionally, when uncoupled ion flow is introduced to our sugar slip model, the width of the detoxification regime is unaffected as the ion leak increases (green region in panels D-E). In fact, the effects of Na^+^-slip and sugar slip appear to be orthogonal with the former causing a leftward shift of the current-voltage curves and the latter causing a downward shift (compare curves in panel A with panel D).

Biophysically, ion leak alone causes a decrease in the ion:substrate stoichiometry, reducing the overall sugar and toxin pumping rates by identical amounts since this leak flux is independent of either substrate. This reduction in pumping pushes the kinetic stall point, where the pumping fluxes go to zero, to more negative voltages (panels A-C). In the presence of sugar slip, when the 4 → 6, 4’ → 6’ and 5 → 7 transitions are allowed, the uncoupled ion leak also pushes the current-voltage curves to the left, but the enhanced selectivity in panel D caused by the branched pathway of the sugar slip cycle persists because both curves shift in unison.

### Identification of toxic compounds that may exploit SGLT to enter the cell

**Fig 8:**
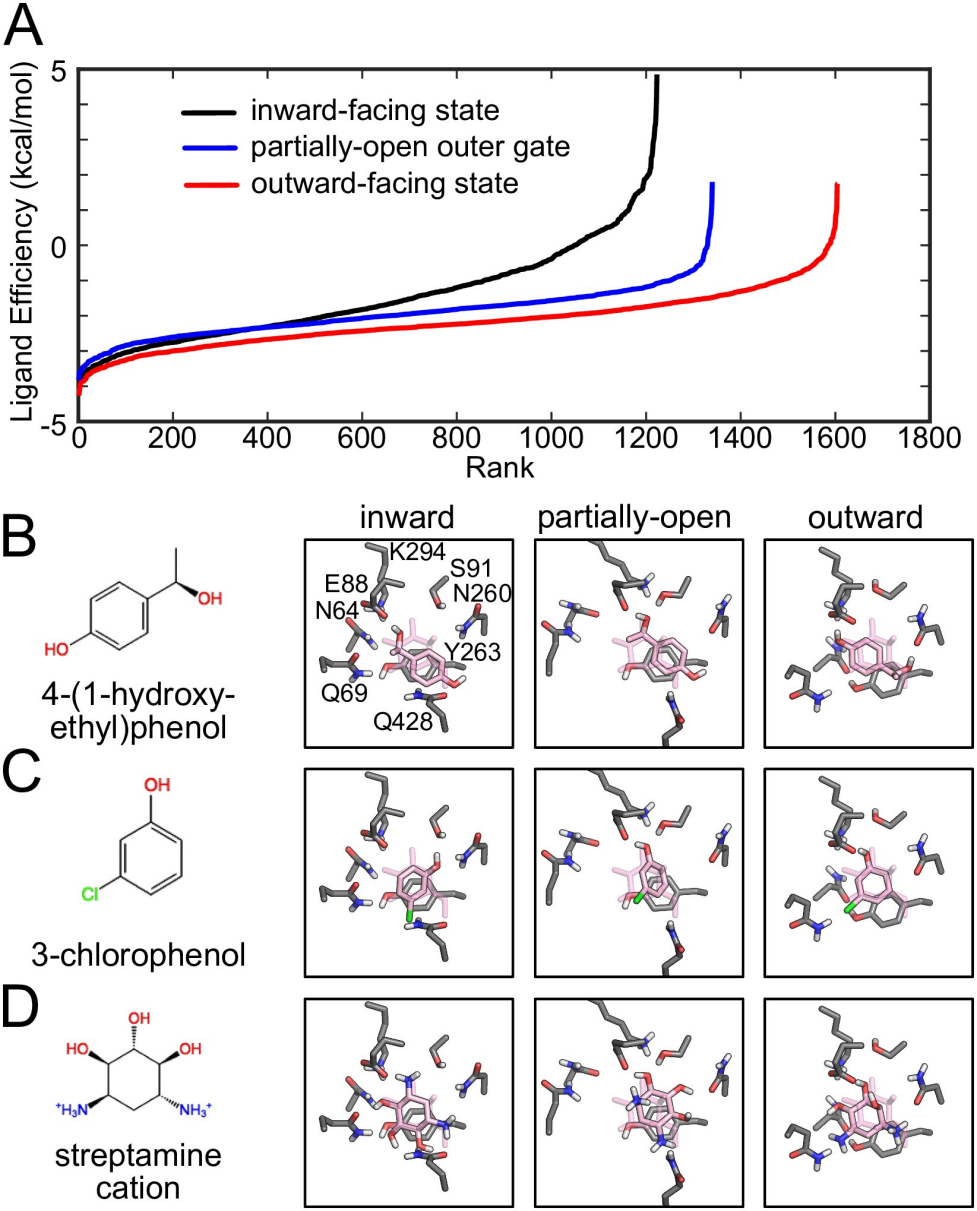
Virtual screening of toxic compounds and sugar analogues. **A)** Ranked ligand efficiency in the three different protein conformations: inward (black), partially-open outer gate (blue), and outward (red). These curves were used to identify the top 200 compounds for each screen to undergo visual inspection. The efficiency parameter for each molecule is normalized by its molecular weight. **B-D)** Four compounds retain a conserved binding mode within all protein conformations: 4-(1-hydroxyl-ethyl)phenol **(B)**, 3-chlorophenol **(C)**, and streptamine **(D)**. The 2D sketch of each chemical is shown in the far left column, while the other columns show the binding poses of each chemical in the inward, partially-open outer gate, and outward conformations, respectively. The interacting protein residues are shown in gray (carbon atoms)/red (oxygens)/blue (nitrogen atoms)/white (polar hydrogen atoms). The galactose pose solved in the holo vSGLT structure (PDB ID: 3DH4) is rendered in transparent pink for reference.

As noted above, there are several examples in the literature of transporters that have difficulty distinguishing correct from incorrect substrates, and one reason for this lack of discrimination may be that weak binding affinities are required for high turnover rates. Thus, we sought to identify toxic galactose analogues capable of binding to vSGLT that could be toxic to *Vibrio parahaemolyticus* if transported. We searched public chemical databases (ZINC and ChEBI) and filtered by physico-chemical properties that maintain the interaction landscape of galactose in the vSGLT binding pocket, as described in the Model and Methods. Each potential compound was docked into three different vSGLT conformations with different degrees of outer gate opening. Next, we re-ranked the docked dataset with MM/GBSA-based rescoring to attempt to discriminate between binders and non-binders and plotted the ligand efficiency for each molecule against the inward-facing (black curve), partially-open outer gate (blue curve), and outward-facing state (blue curve) in rank order (Fig 8A). Among the top 200 compounds in each set, we identified 17 molecules common to all three; and we reasoned that these may be excellent candidates for potential transport by vSGLT. We visually inspected all 17 compounds and identified 3 chemicals that retained most of the galactose interactions observed in the galactose bound inward-facing vSGLT structure [18].

The chemical structures of these molecules along with their putative binding poses are pictured in Fig 8B–8D. Each toxin is represented by solid sticks colored pink/red/white with the original galactose shown in transparent pink for reference. The key protein binding site residues are depicted by gray/red/blue/white sticks and labeled in panel B for the inward-facing state. The first two molecules in panels B (4-(1-hydroxyl-ethyl)phenol) and C (3-chlorophenol) are phenols—compounds that form highly reactive radicals and are widely utilized in plants as secondary metabolites for protection against micro-organisms [32]. The aromatic rings of the phenols make *π*-*π* interactions with Y263 in the highly favorable parallel displaced fashion, a common feature observed in sugar binding sites. Additionally, 4-(1-hydroxyl-ethyl)phenol makes hydrogen-bonding interactions with the known binding site residues E88, N260, and Q428 (only in inward-facing state), while the hydroxyl of 3-chlorophenol engages E88 and S91 in several states and the chloride interacts with the carboxamide of Q428 (inward), Q69 (partially-open and outward). In panel D, the compound streptamine is pictured. This chemical is a common moiety found in several antibiotics, such as *streptomycin* and *bluensomycin*, that bind to the 30 S component of the bacterial ribosome, and streptamine containing agents can cause mRNA misreading [33]. The antibiotic engages its aliphatic ring in stacking with Y263 while reproducing most of the galactose hydrogen bonding interactions observed in all three transporter conformations with its 3 hydroxyl groups. The binding mode of streptamine, as well as several other chemicals discussed here, changes between all protein conformations, and we have observed this behavior previously for sugar as well [17, 34].

### Conclusion

We began with a dilemma: Secondary active transporters bind their substrates weakly, often at relatively low concentrations. How then do they achieve the high selectivity required to avoid transporting lethal quantities of toxic analogs that are structurally similar to their cognate substrates? Clearly this would create strong evolutionary pressure towards a mechanism for the enhanced selectivity of transport. To answer this question, we then turned towards a common feature of known proofreading and editing mechanisms [13–15] noting that the occasional ejection of the correct substrate, a false positive, was an essential feature of proofreading mechanisms. Indeed, Fersht’s cost-selectivity equation demonstrates that enhanced selectivity requires the rejection of some correct substrate [16].

It often goes unquestioned that transporters only transport their cognate substrates, and they do it with fixed coupling ratios or stoichiometries. This notion precludes the need for enhanced selectivity, while also suggesting that ejection of the correct substrate or slip cannot occur. However, there is emerging evidence suggesting that transporters do not always behave as perfect machines, and that they can suffer from imperfections in coupling mechanisms [30], transport toxic [11] or unexpected substrates [25, 35, 36], and exhibit complex transport pathways [37]. Our vSGLT simulations add to this growing list [17], as we observed rejection of the correct substrate in a manner that costs energy—loss of Na^+^ down its gradient (Fig 1). Based on these observations, we developed a kinetic model of vSGLT that included slip for both a correct substrate (sugar) and an incorrect substrate (toxin) to explore the role of proofreading in transport.

Unlike previous proofreading studies, our kinetic model is fully reversible, as it must be to account for the experimentally observed properties of secondary active transporters. We were able to show that recasting the selectivity, cost, and other salient quantities in terms of flows, rather than rate constants, allowed us to generalize Fersht’s cost-selectivity equation and prove that transport systems could achieve increased selectivity in the presence of slip (Eq 4). However, we stumbled across a novel result—proofreading for a reversible system can lead to unbounded selectivity, which to our knowledge has gone unappreciated in discussions of enhanced selectivity. The unbounded selectivity occurs because the toxin inward flow becomes zero at a driving force that is different from the value at which the sugar pumping rate goes to zero. This is a kinetic phenomenon. Additionally, there then exists a set of conditions under which the toxin is removed from the cell while the correct substrate continues to be imported, which we call the detoxification regime. While this regime is narrow and close to thermodynamic stall in a model with weak single-point discrimination, it can be much broader if there are two-points of discrimination (Fig 6).

Our analysis has been limited to specific substrate/ion conditions for two transport schemes shown in Fig 3 and S2 Fig. First, we only considered a situation in which the sugar and toxin concentrations are high inside the cell compared to the extracellular environment, and when toxin is pumped out during detoxification, it is flowing down its concentration gradient. These conditions were not meant to be physiological, but rather secondary active transporters such as vSGLT pump sugars into the cell (as considered here), and we wanted to keep the toxin conditions the same to make a fair comparison between substrates. We imagine that there are conditions for this model, or other kinetic models, in which the detoxification regime can actively pump toxin out of the cell *against* its concentration gradient, and we intend to explore this possibility in the future. Second, our analysis has been restricted in another sense in that we only investigate two models with a limited exploration of parameter space. Indeed, in a separate work we present a Monte Carlo-based method to explore a much larger parameter space of molecular machines [38]. Rather, here we have shown that a specific model based on the known *in vitro* and *in silico* behavior of SGLT’s shows enhanced selectivity behavior.

Finally, given the challenges inherent in studying membrane transport, we wanted to end by explicitly outlining how our theoretical predictions might be tested experimentally on vSGLT or any other membrane transporter. We suggest that vSGLT will transport some of the small molecules in Fig 8B–8D along with other top ranked compounds from our docking study. First, these compounds will be tested with proteoliposome uptake assays to determine if they inhibit normal transport of radiolabeled galactose. Inhibition could result from simply binding to vSGLT or from competitive transport. Radiolabeled versions of the most potent inhibitors will then be created, and we will use the recent method devised by the Mindell lab for electrogenic transporters to map out the uptake rate as a function of imposed voltage [39]. This will be done for labeled galactose, labeled toxin, and combinations of both. These studies will identify the voltage at which the galactose and toxin pumping rates go to zero (the reversal potential) just as in Figs 4A and 6, where a separation in reversal potentials indicates increased selectivity. Thus, these experiments will directly test our theory that vSGLT may exhibit enhanced selectivity via a proofreading mechanism, and if it does, additional biophysical experiments would then be required to tease apart the actual mechanistic details.

## Model and methods

Here, we provide technical details relating to the construction and numerical solution of the kinetics schemes discussed throughout the manuscript. Also, we outline the computational scheme for identifying toxic small molecules that potentially bind to the sugar transporter vSGLT. Additional technical details can be found in the Supporting Information (S1 Text).

### Formulation and numeric solution of the kinetic model

The flux from state *i* to *j* for any two connected states in the model is given by:

$$\begin{array}{c} J_{i,j}=k_{i,j}C_{i}-k_{j,i}C_{j}, \end{array}$$ (5)

where *k*_*i*,*j*_ and *k*_*j*,*i*_ are the pseudo first order rate constants for the forward and reverse transitions, respectively, and *C*_*i*_ and *C*_*j*_ are the time-dependent transporter probabilities for states *i* and *j*, respectively. We denote true rate constants with a 0 superscript, thus $k_{i,j}=k_{i,j}^{0}{\left[L\right]}_{out/in}$, where ${\left[L\right]}_{out/in}$ is the extracellular (out) or cytoplasmic (in) concentration of a given ligand (toxin, sugar, Na^+^) for those transitions that involve ligand exchange with solution. For any given solution of the model, sugar, toxin, and ion concentration values as well as the membrane potential (*V*) are held constant. We use Eyring rate theory to describe the dependence of rate constants on membrane potential (*V*):

$$\begin{array}{c} k_{ij}=k_{ij}^{0}\left[L\right]exp\left(-\epsilon_{ij}FV/RT\right), \end{array}$$ (6)

where *ϵ*_*ij*_ is the net charge movement for the *i* → *j* transition and *ϵ*_*ij*_ = −*ϵ*_*ji*_. *F*, *R*, and *T* have their usual physicochemical meanings [40].

In order to cast the kinetics into an energetic framework, which is easier to interpret, we express all equilibrium constants (*K*_*ij*_) and all rate constants (*k*_*ij*_) by dimensionless standard free energy ($\Delta G_{ij}^{0}$) and dimensionless transition state interaction energy terms ($\Delta G_{ij}^{‡}$), respectively:

$$\begin{array}{ccc} K_{ij} & \equiv & exp(-\Delta G_{ij}^{0})\\ k_{ij}^{0} & \equiv & exp(-\Delta G_{ij}^{‡})\\ k_{ji}^{0} & = & \frac{k_{ij}^{0}}{K_{ij}}=exp\left(-\left(\Delta G_{ij}^{‡}-\Delta G_{ij}^{0}\right)\right) \end{array}$$ (7)

Toxin pathways are defined below (see also Fig 3) such that they are symmetric with those of the sugar. The energy values on the toxin pathways are expressed in terms of differences compared to the corresponding values on the sugar pathways as follows:

$$\begin{array}{ccc} \Delta G_{3^{\prime }4^{\prime }}^{0} & = & \Delta G_{34}^{0}+\Delta \Delta G_{3^{\prime }4^{\prime }}^{0}\\ \Delta G_{3^{\prime }4^{\prime }}^{‡} & = & \Delta G_{34}^{‡}+\Delta \Delta G_{3^{\prime }4^{\prime }}^{‡} \end{array}$$ (8)

Finally, detailed balance around all complete cycles is then enforced with the following relations:

$$\begin{array}{ccc} \Delta G_{54}^{0} & = & \Delta G_{12}^{0}+\Delta G_{23}^{0}+\Delta G_{34}^{0}+\Delta G_{51}^{0}\\ \Delta \Delta G_{54^{\prime }}^{0} & = & \Delta \Delta G_{23^{\prime }}^{0}+\Delta \Delta G_{3^{\prime }4^{\prime }}^{0}\\ \Delta G_{57}^{0} & = & \Delta G_{54}^{0}+\Delta G_{46}^{0}-\Delta G_{76}^{0}\\ \Delta \Delta G_{76^{\prime }}^{0} & = & \Delta \Delta G_{54^{\prime }}^{0}+\Delta \Delta G_{4^{\prime }6^{\prime }}^{0} \end{array}$$ (9)

Differential equations were solved using the stiff solver ode15s with default parameters in Matlab R2015a (The Mathworks Inc., Nantick, MA). Systems were run to steady state, and the final values were used to construct all graphs. All model parameters and additional equations can be found in the Supporting Information.

### Virtual screening

A dataset for galactose analogues and putative toxic compounds was generated by searching the ChEBI [41] and ZINC [42] databases using the Small Molecules Drug Discovery Suite version 2017-4 [43] for all steps in the docking procedure. The ChEBI search terms were: carcinogens, antibiotics, toxic compounds and phenols; and this initial list was subsequently filtered again for the following features: number of heavy atoms ≤ 37, polar surface area < 274 Å^2^, and number of rotatable bonds ≤ 11. In the ZINC database, we searched for galactose analogues with 60% similarity score, and we applied the following filters: molecular weight ≤ 270 Da and number of rotatable bonds ≤ 7. In addition, we required all compounds from both databases to have one aromatic/aliphatic ring and at least one hydrogen-bond donor/acceptor group. The ZINC search yielded 1,224 galactose analogues including alternative tautomers and stereoisomers, while the ChEBI returned 341 molecules which we subjected to chemical expansion with *Epik*-v4.2 [44] to generate 871 states. The final docking dataset contained 2,095 molecules, whose partial charges were assigned with *LigPrep* [45]. This dataset was then filtered and prepared in the ready-to-dock format.

We docked each molecule into the orthosteric binding site of three different vSGLT protein conformations: the inward-facing X-ray structure [18], an outward-facing homology model of vSGLT [46] based on the closely related sialic acid transporter SiaT [34], and a snapshot from a simulation of the inward-facing state in which the outer gate partially-opened [17]. Prior to docking, all the protein conformations were aligned and the galactose position of the inward-facing structure was used to center a 17 × 17 × 17 Å box for docking grid generation. Restraints were applied to the hydrogen bond acceptor and donor groups of the sugar binding site residues (caboxyl group of E88, hydroxyl of S91, amine of K294, and carboxamide of N64, Q68, and N260) to reproduce the hydrogen bonding network observed in the galactose-bound structure. Additionally, the carboxamide of Q428 was restrained only in the inward and partially-open states, since it does not contact the sugar in the outward-open conformation [47]. Rigid docking was performed with *Glide XP* keeping the protein fixed while the ligand was allowed to freely rotate around its rotatable bonds. Halogens were treated as hydrogen-bond acceptors and aromatic hydrogens as hydrogen-bond donors. Final poses were only considered if they satisfied at least two of the hydrogen bond restraints. After docking, the protein-ligand complexes were minimized with the MM/GBSA protocol [48] using VSGB solvent and an implicit membrane, which is a low dielectric slab-shaped region [49]. The final set was ranked based on the MM/GBSA ligand efficiency parameter to normalize by molecular weight, and the top 10 − 20% of the rescored dataset was visually inspected.

## Supporting information

S1 Text Kinetic model. (PDF)

S2 Text Derivation of proofreading equations. (PDF)

S1 FigDetailed description of sugar cycles in the kinetic model with slip.The upper three panels indicate the 3 modes of sugar transport based on the full kinetic model in Fig 3 of the main text. The bottom three panels indicate the mathematical formula for $J^{pump}$, $J^{bind}$, and $J^{slip}$ and the corresponding kinetic transitions from the model. While not shown, the cycles and flows for toxin are analogous to the ones here for toxin.(TIF)

S2 Fig Six State Model. Sodium slip was introduced into our model via the addition of state 8, and the associated transitions, as discussed in S1 Text and/or as can be seen by comparison with the model shown in Fig 3. The modified kinetic model includes two sub cycles that allow sugar slip from the cytoplasmic to the extracellular compartment with no net transport of sodium ion. One of these is 8 → 4 → 3 → 2 → 8. Note that the sodium ion remains bound to the transporter during this cycle which is unique to this model. The other sub cycle has an analogous sub cycle in Fig 3 (see also S1 Fig), specifically it is 8 → 4 → 6 → 7 → 5 → 8. Here, although sodium dissociates from state 6 in the 6 → 7 transition, it rebinds from the same cytoplasmic side in the 5 → 8 transition.
(TIF)

S1 Table Transition energies along sugar cycles. (PDF)

S2 Table Transition energies along toxin cycles. (PDF)

S3 Table Voltage dependence of molecular transitions. (PDF)

S1 Model 5 state kinetic model with substrate slip. The ordinary differential equations (ODE) model created with Matlab. This model corresponds to the canonical 5 state (along normal sugar transport cycle) cartoon model in Fig 3 with a substrate slip. The file format is native Matlab, and it can be opened and run with Matlab. The model is run by opening Matlab, navigating to the directory containing the code (file_name.m file) and typing the name of the file (without “.m”) at the command prompt. When run, the code will generate 3 figures: 1) the first figure corresponds to Fig 4 from the main text, 2) the second figure corresponds to Fig 5 in the main text, and 3) the final the third figure corresponds to Fig 6 in the main text.
(M) Click here for additional data file.

S2 Model 6 state kinetic model with substrate slip and Na^+^-leak. The ordinary differential equations (ODE) model created with Matlab. This model corresponds to a 6 state (along normal sugar transport cycle) cartoon model in S2 Fig with a substrate slip and Na^+^ leak. The file format is native Matlab, and it can be opened and run with Matlab. The model is run by opening Matlab, navigating to the directory containing the code (file_name.m file) and typing the name of the file (without “.m”) at the command prompt. When run, the code will generate 1 figure corresponding to Fig 7 in the main text.
(M) Click here for additional data file.
