# Supplementary material for: A kinetic mechanism for enhanced selectivity of membrane transport
Source: PLoS Comput Biol. 2020 Jul 2;16(7):e1007789. doi: 10.1371/journal.pcbi.1007789 (PMC7331977; doi:10.1371/journal.pcbi.1007789)
Supplement: S1 Text — (PDF) [file pcbi.1007789.s001.pdf]

S1 Text. Kinetic Model

The full set of differential equations corresponding to the model in Fig 3 is:

$$\begin{aligned}
\frac{dC_1}{dt} &= -(k_{12}+k_{15})C_1 + k_{21}C_2 + k_{51}C_5 \\
\frac{dC_2}{dt} &= k_{12}C_1 - (k_{21}+k_{23}+k_{23'})C_2 + k_{32}C_3 + k_{3'2}C_{3'} \\
\frac{dC_3}{dt} &= k_{23}C_2 - (k_{32}+k_{34})C_3 + k_{43}C_4 \\
\frac{dC_{3'}}{dt} &= k_{23'}C_2 - (k_{3'2}+k_{3'4'})C_{3'} + k_{4'3'}C_{4'} \\
\frac{dC_4}{dt} &= k_{34}C_3 - (k_{43}+k_{45}+k_{46})C_4 + k_{54}C_5 + k_{64}C_6 \\
\frac{dC_{4'}}{dt} &= k_{3'4'}C_{3'} - (k_{4'3'}+k_{4'5}+k_{4'6'})C_{4'} + k_{54'}C_5 + k_{6'4'}C_{6'} \\
\frac{dC_5}{dt} &= k_{15}C_1 + k_{45}C_4 + k_{4'5}C_{4'} - (k_{51}+k_{54}+k_{54'}+k_{57})C_5 + k_{75}C_7 \\
\frac{dC_6}{dt} &= k_{46}C_4 - (k_{64}+k_{67})C_6 + k_{76}C_7 \\
\frac{dC_{6'}}{dt} &= k_{4'6'}C_{4'} - (k_{6'4'}+k_{6'7})C_{6'} + k_{76'}C_7 \\
\frac{dC_7}{dt} &= k_{57}C_5 + k_{67}C_6 + k_{6'7}C_{6'} - (k_{75}+k_{76}+k_{76'})C_7, \qquad (1)
\end{aligned}$$

where C_i is the occupancy probability for state i, and $\sum C_i = 1$. Transitions between states C_i and C_j are represented by rate constants k_{ij}: $C_i \rightarrow C_j$. The specific relations for the rate constants are given in terms of the underlying energetics (ΔG, and $\Delta\Delta G$ values) :

$$
\begin{aligned}
k_{12} &= [Na^+]^n exp(-(\Delta G^{\ddagger}_{12} + \epsilon_{12} FV/RT)) \\
k_{21} &= exp(-(\Delta G^{\ddagger}_{12} - \Delta G^{0}_{12} + \epsilon_{21} FV/RT)) \\
k_{23} &= [\mathcal{S}]_{out} exp(-(\Delta G^{\ddagger}_{23} + \epsilon_{23} FV/RT)) \\
k_{32} &= exp(-(\Delta G^{\ddagger}_{23} - \Delta G^{0}_{23} + \epsilon_{32} FV/RT)) \\
k_{23'} &= [\mathcal{W}]_{out} exp(-(\Delta G^{\ddagger}_{23} + \Delta\Delta G^{\ddagger}_{23'})) \\
k_{3'2} &= exp(-(\Delta G^{\ddagger}_{23} + \Delta\Delta G^{\ddagger}_{23'} - \Delta G^{0}_{23} - \Delta\Delta G^{0}_{23'})) \\
k_{34} &= exp(-(\Delta G^{\ddagger}_{34} + \epsilon_{34} FV/RT)) \\
k_{43} &= exp(-(\Delta G^{\ddagger}_{34} - \Delta G^{0}_{34} + \epsilon_{43} FV/RT)) \\
k_{3'4'} &= exp(-(\Delta G^{\ddagger}_{34} + \Delta\Delta G^{\ddagger}_{3'4'} + \epsilon_{34} FV/RT)) \\
k_{4'3'} &= exp(-(\Delta G^{\ddagger}_{34} + \Delta\Delta G^{\ddagger}_{3'4'} - \Delta G^{0}_{34} - \Delta\Delta G^{0}_{3'4'} + \epsilon_{43} FV/RT)) \\
k_{45} &= exp(-(\Delta G^{\ddagger}_{54} - \Delta G^{0}_{54} + \epsilon_{45} FV/RT)) \\
k_{54} &= [Na^+]^n_{in} [\mathcal{S}]_{in} exp(-(\Delta G^{\ddagger}_{54} + \epsilon_{45} FV/RT)) \\
k_{4'5} &= exp(-(\Delta G^{\ddagger}_{54} + \Delta\Delta G^{\ddagger}_{54'} - \Delta G^{0}_{54} - \Delta\Delta G^{0}_{54'} + \epsilon_{45} FV/RT)) \\
k_{54'} &= [Na^+]^n_{in} [\mathcal{W}]_{in} exp(-(\Delta G^{\ddagger}_{54} + \Delta\Delta G^{\ddagger}_{54'} + \epsilon_{54} FV/RT)) \\
k_{15} &= exp(-(\Delta G^{\ddagger}_{51} - \Delta G^{0}_{51} + \epsilon_{15} FV/RT)) \\
k_{51} &= exp(-(\Delta G^{\ddagger}_{51} + \epsilon_{51} FV/RT)) \\
k_{46} &= exp(-\Delta G^{\ddagger}_{46}) \\
k_{64} &= exp(-(\Delta G^{\ddagger}_{46} - \Delta G^{0}_{46})) \\
k_{4'6'} &= exp(-(\Delta G^{\ddagger}_{46} + \Delta\Delta G^{\ddagger}_{4'6'})) \\
k_{6'4'} &= exp(-(\Delta G^{\ddagger}_{46} + \Delta\Delta G^{\ddagger}_{4'6'} - \Delta G^{0}_{46} - \Delta\Delta G^{0}_{4'6'})) \\
k_{76} &= [Na^+]^n_{in} [S]_{out} exp(-\Delta G^{\ddagger}_{76}) \\
k_{67} &= exp(-(\Delta G^{\ddagger}_{76} - \Delta G^{0}_{76})) \\
k_{76'} &= [Na^+]^n_{in} [\mathcal{W}]_{out} exp(-(\Delta G^{\ddagger}_{76} + \Delta\Delta G^{\ddagger}_{76'})) \\
k_{6'7} &= exp(-(\Delta G^{\ddagger}_{76} + \Delta\Delta G^{\ddagger}_{76'} - \Delta G^{0}_{76} - \Delta\Delta G^{0}_{76'})) \\
k_{57} &= exp(-\Delta G^{\ddagger}_{57}) \\
k_{75} &= exp(-(\Delta G^{\ddagger}_{57} - \Delta G^{0}_{57})), \qquad (2)
\end{aligned}
$$

where $[\mathcal{S}]$ is the sugar substrate concentration on the inside or the outside of the cell, $[\mathcal{W}]$ is similarly the concentration of the wrong substrate (toxin), and $[Na^+]$ is the sodium concentration on both sides of the membrane and n is 1 for vSGLT.

Addition of Na$^+$ slip

To model Na$^+$ slip, as shown in S2 Fig, we added an additional state along the standard transport cycle, which call call state 8 and corresponds to the inward-facing substrate bound state. We removed the rates connecting state 4 to state 5 directly and added intermediate steps corresponding to $\{k_{48}, k_{84}, k_{85}, k_{58}, k_{4'8}, k_{84'}\}$. This additional step makes it possible to explicitly model the Na$^+$ leak from outward-facing sodium bound to inward-facing sodium bound states with the addition of two new rates k_{28} and k_{82} shared by both the toxin and sugar cycles. The following changes to the differential equations are:

$$\begin{aligned}
\frac{dC_2}{dt} &= k_{12}C_1 - (k_{21} + k_{23} + k_{23'} + k_{28})C_2 + k_{32}C_3 + k_{3'2}C_{3'} + k_{82}C_8 \\
\frac{dC_4}{dt} &= k_{34}C_3 - (k_{43} + k_{46} + k_{48})C_4 + k_{84}C_8 + k_{64}C_6 \\
\frac{dC_{4'}}{dt} &= k_{3'4'}C_{3'} - (k_{4'3'} + k_{4'6'} + k_{4'8})C_{4'} + k_{84'}C_8 + k_{6'4'}C_{6'} \\
\frac{dC_5}{dt} &= k_{15}C_1 - (k_{51} + k_{57} + k_{58})C_5 + k_{75}C_7 + k_{85}C_8 \\
\frac{dC_8}{dt} &= k_{45}C_4 + k_{4'8}C_{4'} + k_{58}C_5 - (k_{82} + k_{84} + k_{84'} + k_{85})C_8
\end{aligned} \quad (3)$$

and we make the following changes to the rate constants:

$$\begin{aligned}
k_{45} &= k_{54} = k_{4'5} = k_{54'} = 0 \\
k_{48} &= exp(-(\Delta G^{\ddagger}_{48} - \Delta G^{0}_{48})) \\
k_{84} &= [\mathcal{S}]_{in} exp(-\Delta G^{\ddagger}_{48}) \\
k_{4'8} &= exp(-(\Delta G^{\ddagger}_{48} + \Delta\Delta G^{\ddagger}_{4'8} - \Delta G^{0}_{48} - \Delta\Delta G^{0}_{4'8})) \\
k_{84'} &= [\mathcal{W}]_{in} exp(-(\Delta G^{\ddagger}_{48} + \Delta\Delta G^{\ddagger}_{4'8})) \\
k_{85} &= exp(-(\Delta G^{\ddagger}_{58} - \Delta G^{0}_{58} + \epsilon_{85}FV/RT)) \\
k_{58} &= [Na^{+}]^{n}_{in} exp(-(\Delta G^{\ddagger}_{58} + \epsilon_{58}FV/RT)) \\
k_{28} &= exp(-(\Delta G^{\ddagger}_{28} - \Delta G^{0}_{28} + \epsilon_{28}FV/RT)) \\
k_{82} &= exp(-(\Delta G^{\ddagger}_{28} + \epsilon_{82}FV/RT))
\end{aligned} \quad (4)$$

where $\epsilon_{85} = \epsilon_{45}$ from the model with no Na^{+}-leak and $\epsilon_{28} = \epsilon_{23} + \epsilon_{34}$ (see S3 Table for values) and all energies can be found in S1-S2 Table. We constructed the rates for the new state 8 ($\{k_{48}, k_{84}, k_{85}, k_{58}, k_{4'8}, k_{84'}\}$) such that without Na^{+}-leak the model gave a very similar overall current-voltage curve to the model without state 8. Results based on this model are shown in Fig 7 and are discussed in the main text. The variable ion leak rates in Fig 7 are $k_{28} =$ 0, 100 and 200 s^{-1}, and the corresponding energy values are $\Delta G^{\ddagger}_{28} =$ 0, -3.11, and -3.11 k_BT and $\Delta G_{28} =$ 0, -4.61, and -5.30 k_BT, respectively.
