# Supplementary material for: A kinetic mechanism for enhanced selectivity of membrane transport
Source: PLoS Comput Biol. 2020 Jul 2;16(7):e1007789. doi: 10.1371/journal.pcbi.1007789 (PMC7331977; doi:10.1371/journal.pcbi.1007789)
Supplement: S2 Text — (PDF) [file pcbi.1007789.s002.pdf]

S2 Text. Derivation of proofreading equations

The flow of substrates in the model are given by the following equations:

$$\begin{aligned}
\mathcal{J}_{\mathcal{S}}^{pump} &\equiv \mathcal{J}_{\mathcal{S},4\to5} = k_{45}C_4 - k_{54}C_5 \\
\mathcal{J}_{\mathcal{S}}^{slip} &\equiv \mathcal{J}_{\mathcal{S},6\to7} = k_{67}C_6 - k_{76}C_7 \\
\mathcal{J}_{\mathcal{S}}^{bind} &\equiv \mathcal{J}_{\mathcal{S},2\to3} = k_{23}C_2 - k_{32}C_3,
\end{aligned} \quad (5)$$

where $\mathcal{S}$ indicates sugar. Similarly, the full set of flows of the wrong substrate (the toxin) is given by:

$$\begin{aligned}
\mathcal{J}_{\mathcal{W}}^{pump} &\equiv \mathcal{J}_{\mathcal{W},4'\to5} = k_{4'5}C_{4'} - k_{54'}C_5 \\
\mathcal{J}_{\mathcal{W}}^{slip} &\equiv \mathcal{J}_{\mathcal{W},6'\to7} = k_{6'7}C_{6'} - k_{76'}C_7 \\
\mathcal{J}_{\mathcal{W}}^{bind} &\equiv \mathcal{J}_{\mathcal{W},2\to3'} = k_{23'}C_2 - k_{3'2}C_{3'},
\end{aligned} \quad (6)$$

where $\mathcal{W}$ indicates toxin.

We now construct the classic cost-selectivity equation proposed by Fersht [1] by recasting the classical enzymological specificity constant ratio (f) in terms of flows:

$$f \equiv \frac{\mathcal{J}_{\mathcal{S}}^{bind}}{\mathcal{J}_{\mathcal{W}}^{bind}} \frac{\Delta[\mathcal{W}]}{\Delta[\mathcal{S}]}, \quad (7)$$

where $\Delta[\mathcal{W}] = [\mathcal{W}_{out}] - [\mathcal{W}_{in}]$ and $\Delta[\mathcal{S}] = [\mathcal{S}_{out}] - [\mathcal{S}_{in}]$. In the absence of slip, f becomes f_{noslip}, and it is easy to show that it is the conventional ratio of $\frac{k_{cat}}{k_M}$ (see Ref. [1]):

$$f_{noslip} = (k_{cat}/K_M)_{\mathcal{S}}/(k_{cat}/K_M)_{\mathcal{W}}, \quad (8)$$

The quantity f_{noslip} becomes a baseline for enhanced selectivity, since there can be no enhanced selectivity without slip. Next, Fersht defined two additional ratios, f' and f'', that bear directly on the selectivity. Here, we redefine them in terms of flows:

$$\begin{aligned}
f' &\equiv \frac{\mathcal{J}_{\mathcal{S}}^{pump}}{\mathcal{J}_{\mathcal{W}}^{pump}} \\
f'' &\equiv \frac{\mathcal{J}_{\mathcal{W}}^{slip}}{\mathcal{J}_{\mathcal{S}}^{slip}},
\end{aligned} \quad (9)$$

where f' is the ratio of the successfully pumped flow of sugar to that of toxin ($4 \to 5$ to $4' \to 5$) whereas f'' is the ratio of the slip flow of toxin to that of sugar ($6' \to 7$ to $6 \to 7$, see Fig 3 main text).

The core of Fersht's argument is that there is a price that must be paid for enhanced selectivity [1], and the exact correspondence here is obtained by comparing the efficiency of transport. We do this by defining the ratio of rates for total substrate binding from the extracellular space to successful substrate release to the cytoplasm:

$$
\begin{aligned}
F_{\mathcal{S}} &\equiv \frac{\mathcal{J}_{\mathcal{S}}^{pump}}{\mathcal{J}_{\mathcal{S}}^{pump}+\mathcal{J}_{\mathcal{S}}^{slip}} = \frac{\mathcal{J}_{\mathcal{S}}^{pump}}{\mathcal{J}_{\mathcal{S}}^{bind}} \\
F_{\mathcal{W}} &\equiv \frac{\mathcal{J}_{\mathcal{W}}^{pump}}{\mathcal{J}_{\mathcal{W}}^{pump}+\mathcal{J}_{\mathcal{W}}^{slip}} = \frac{\mathcal{J}_{\mathcal{W}}^{pump}}{\mathcal{J}_{\mathcal{W}}^{bind}},
\end{aligned} \tag{10}
$$

where the formal definition is general, i.e. non-steady state which reduces to the second set of ratios at steady state.

The price for enhanced selectivity is given by the fractional rate of false positive errors, i.e. the rate of rejection of valid substrate (sugar), divided by the rate of binding of valid substrate. Specifically, he defines that fractional rate as the cost ($\mathcal{C}$)which can be recast in terms of flows as follows:

$$
\mathcal{C} \equiv \frac{\mathcal{J}_{\mathcal{S}}^{slip}}{\mathcal{J}_{\mathcal{S}}^{bind}} = \frac{\mathcal{J}_{\mathcal{S}}^{slip}}{\mathcal{J}_{\mathcal{S}}^{pump}+\mathcal{J}_{\mathcal{S}}^{slip}} \tag{11}
$$

Next, we define the selectivity (σ) of transport as:

$$
\sigma \equiv \frac{\mathcal{J}_{\mathcal{S}}^{pump}}{\mathcal{J}_{\mathcal{W}}^{pump}} \frac{\Delta[\mathcal{W}]}{\Delta[\mathcal{S}]}, \tag{12}
$$

where we use σ rather than S, as used by Fersht, due to the extensive use of the letter S throughout. Incorporation of the concentration gradients in Eq. 12 is crucial because non-selective transport ($\sigma = 1$) results in flows that scale linearly with driving force, which would incorrectly be interpreted as selectivity without accounting for the gradients.

Another way of stating Fersht's core argument is that there is an intimate and inescapable relationship between cost and selectivity [1]. That relationship can be seen via two straightforward rearrangements of the quantities already defined, specifically:

$$
\begin{aligned}
f\frac{\Delta[\mathcal{S}]}{\Delta[\mathcal{W}]}\frac{F_{\mathcal{S}}}{F_{\mathcal{W}}} &= \frac{\mathcal{J}_{\mathcal{S}}^{pump}}{\mathcal{J}_{\mathcal{W}}^{pump}} \\
\frac{F_{\mathcal{S}}}{F_{\mathcal{W}}} &= 1 + (f'f'' - 1)\mathcal{C}
\end{aligned} \tag{13}
$$

Hence,

$$
\begin{aligned}
\sigma &= f\frac{F_{\mathcal{S}}}{F_{\mathcal{W}}} \\
\sigma &= f\{1 + (f'f'' - 1)\mathcal{C}\}
\end{aligned} \tag{14}
$$

Eq. 14 is Fersht's cost-selectivity equation, which we have now obtained based on a fully reversible system by recasting the underlying definitions in terms of flows.

References

1. Fersht A. Structure and mechanism in protein science: a guide to enzyme catalysis and protein folding. New York: Macmillan; 1999.
