# Supplementary figures and images for: A kinetic mechanism for enhanced selectivity of membrane transport
Source: PLoS Comput Biol. 2020 Jul 2;16(7):e1007789. doi: 10.1371/journal.pcbi.1007789 (PMC7331977; doi:10.1371/journal.pcbi.1007789)

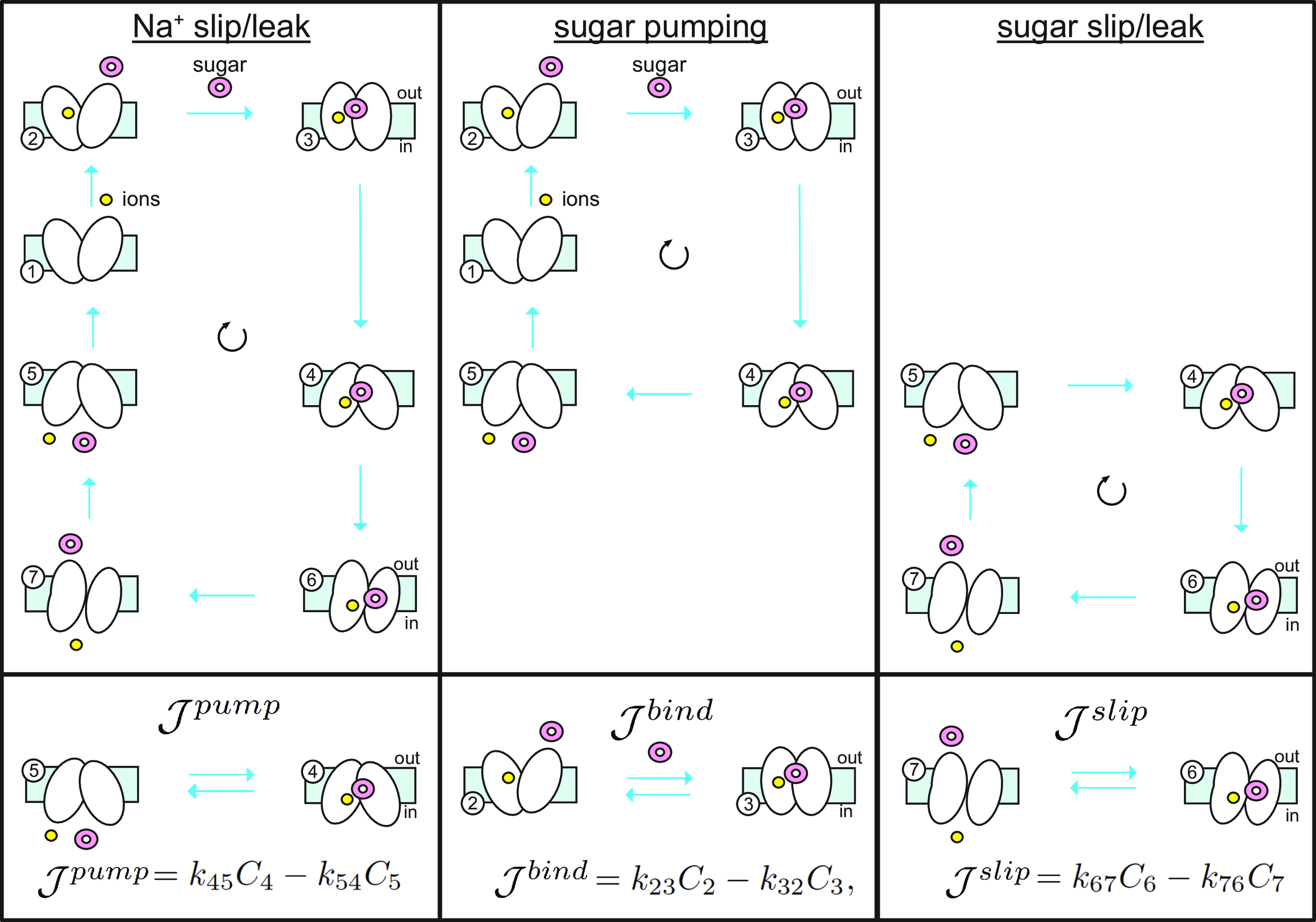

Supplement: S1 Fig — The upper three panels indicate the 3 modes of sugar transport based on the full kinetic model in Fig 3 of the main text. The bottom three panels indicate the mathematical formula for Jpump, Jbind, and Jslip and the corresponding kinetic transitions from the model. While not shown, the cycles and flows for toxin are analogous to the ones here for toxin. (TIF) [file pcbi.1007789.s003.tif]

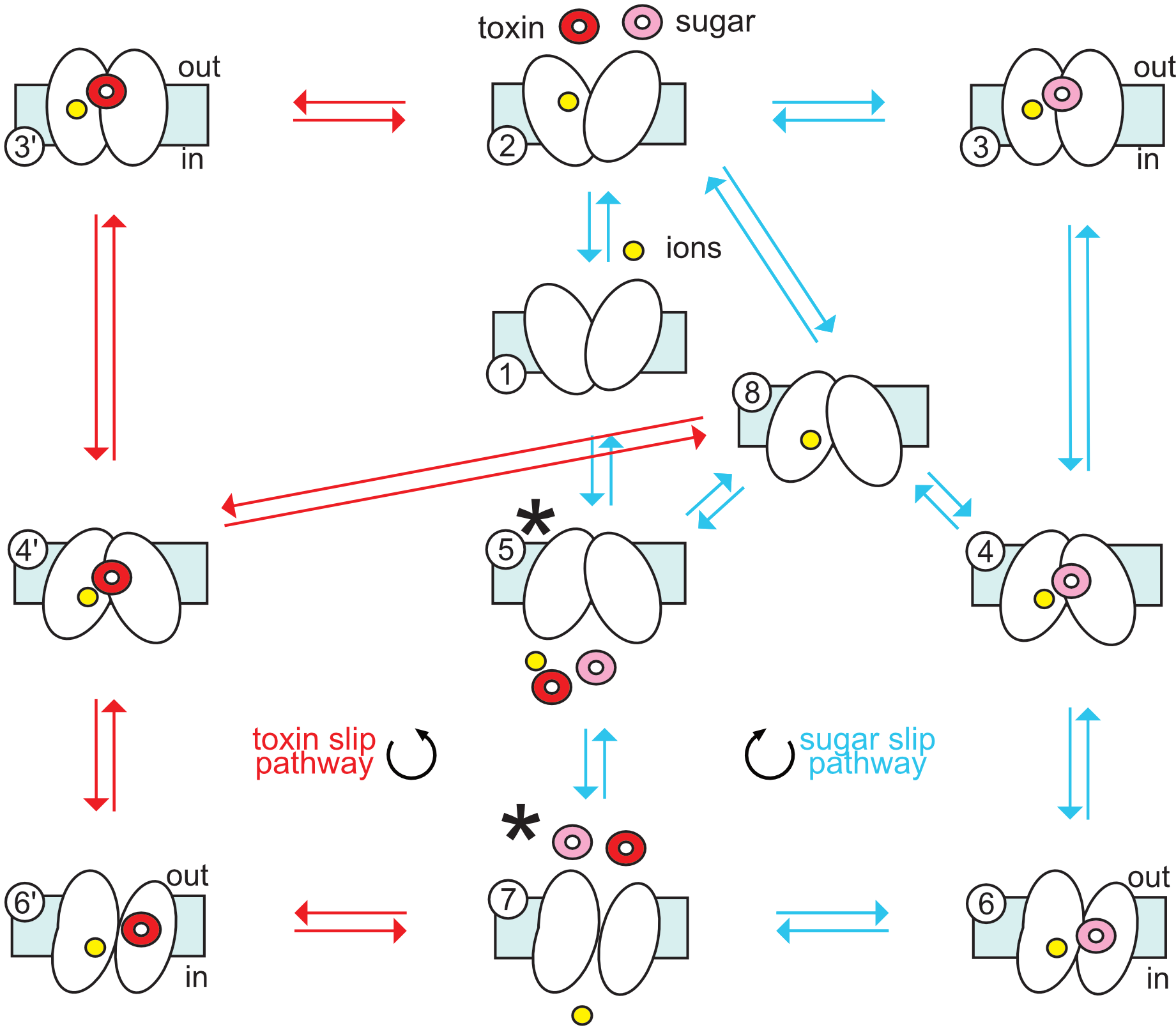

Supplement: S2 Fig — Sodium slip was introduced into our model via the addition of state 8, and the associated transitions, as discussed in S1 Text and/or as can be seen by comparison with the model shown in Fig 3. The modified kinetic model includes two sub cycles that allow sugar slip from the cytoplasmic to the extracellular compartment with no net transport of sodium ion. One of these is 8 → 4 → 3 → 2 → 8. Note that the sodium ion remains bound to the transporter during this cycle which is unique to this model. The other sub cycle has an analogous sub cycle in Fig 3 (see also S1 Fig), specifically it is 8 → 4 → 6 → 7 → 5 → 8. Here, although sodium dissociates from state 6 in the 6 → 7 transition, it rebinds from the same cytoplasmic side in the 5 → 8 transition. (TIF) [file pcbi.1007789.s004.tif]
