# Supplementary material for: A kinetic mechanism for enhanced selectivity of membrane transport
Source: PLoS Comput Biol. 2020 Jul 2;16(7):e1007789. doi: 10.1371/journal.pcbi.1007789 (PMC7331977; doi:10.1371/journal.pcbi.1007789)
Supplement: S1 Table — (PDF) [file pcbi.1007789.s005.pdf]

S1 Table. Transition energies along sugar cycles.

Symbol	Energy	Symbol	Energy
ΔG_{12}	-2.18	$\Delta G^{\ddagger}_{12}$	-8.52
ΔG_{23}	-7.72	$\Delta G^{\ddagger}_{23}$	-10.71
ΔG_{34}	0	$\Delta G^{\ddagger}_{34}$	-3.91
ΔG_{45}	7.35	$\Delta G^{\ddagger}_{45}$	-4.61
ΔG_{51}	3.18	$\Delta G^{\ddagger}_{51}$	-3.22
ΔG_{46}	0	$\Delta G^{\ddagger}_{46}$	-3.00
ΔG_{67}	10.53	$\Delta G^{\ddagger}_{67}$	-5.00
ΔG_{57}	3.18	$\Delta G^{\ddagger}_{57}$	-2.00
ΔG_{28}	variable	$\Delta G^{\ddagger}_{28}$	variable
ΔG_{48}	4.61	$\Delta G^{\ddagger}_{48}$	-4.61
ΔG_{85}	2.75	$\Delta G^{\ddagger}_{58}$	-9.21
