# Supplementary material for: A kinetic mechanism for enhanced selectivity of membrane transport
Source: PLoS Comput Biol. 2020 Jul 2;16(7):e1007789. doi: 10.1371/journal.pcbi.1007789 (PMC7331977; doi:10.1371/journal.pcbi.1007789)
Supplement: S2 Table — (PDF) [file pcbi.1007789.s006.pdf]

## S2 Table. Transition energy differences along toxin cycles.

| Symbol | Energy $[k_B T]$ | Symbol | Energy $[k_B T]$ |
|---|---|---|---|
| $\Delta\Delta G_{23'}$ | 0 | $\Delta\Delta G^{\ddagger}_{23'}$ | 0 |
| $\Delta\Delta G_{3'4'}$ | 0 | $\Delta\Delta G^{\ddagger}_{3'4'}$ | 1 |
| $\Delta\Delta G_{4'5}$ | 0 | $\Delta\Delta G^{\ddagger}_{4'5}$ | 0 |
| $\Delta\Delta G_{4'6'}$ | 0 | $\Delta\Delta G^{\ddagger}_{4'6'}$ | variable |
| $\Delta\Delta G_{6'7}$ | 0 | $\Delta\Delta G^{\ddagger}_{6'7}$ | 0 |
| $\Delta\Delta G_{4'8}$ | 0 | $\Delta\Delta G^{\ddagger}_{4'8}$ | 0 |
