# Supplementary material for: A kinetic mechanism for enhanced selectivity of membrane transport
Source: PLoS Comput Biol. 2020 Jul 2;16(7):e1007789. doi: 10.1371/journal.pcbi.1007789 (PMC7331977; doi:10.1371/journal.pcbi.1007789)
Supplement: S3 Table — (PDF) [file pcbi.1007789.s007.pdf]

## S3 Table. Voltage dependence of molecular transitions.

| Symbol | Energy | Symbol | Energy |
|---|---|---|---|
| $\epsilon_{12}$ | 0.2300 | $\epsilon_{21}$ | -0.2300 |
| $\epsilon_{23}$ | -0.1175 | $\epsilon_{32}$ | 0.1175 |
| $\epsilon_{34}$ | -0.1175 | $\epsilon_{43}$ | 0.1175 |
| $\epsilon_{45}$ | 0.1550 | $\epsilon_{54}$ | -0.1550 |
| $\epsilon_{15}$ | -0.3500 | $\epsilon_{51}$ | 0.3500 |
| $\epsilon_{28}$ | -0.2350 | $\epsilon_{82}$ | 0.2350 |
| $\epsilon_{85}$ | 0.1550 | $\epsilon_{58}$ | -0.1550 |
